# Critical complications in cancer patients admitted to the ICU in the era of immunotherapy: recognition, differential diagnosis, and management

**DOI:** 10.3389/fmed.2026.1841937

**Published:** 2026-07-20

**Authors:** Di Wang, Min Yu

**Affiliations:** Department of Critical Care Medicine, The first Hospital of China Medical University, Shenyang, China

**Keywords:** immunotherapy, ICU, toxicity, adverse effects, clinical complications

## Abstract

**Background:**

Immunotherapy has introduced a new spectrum of ICU-relevant complications in patients with cancer, including hyperinflammatory syndromes, neurotoxicity, severe pneumonitis, myocarditis, and overlapping infectious complications.

**Objective:**

To review the major life-threatening complications of cancer immunotherapy from an ICU perspective, focusing on recognition, differential diagnosis, and management.

**Content:**

We discuss the critical illness patterns associated with CAR-T therapy, immune checkpoint inhibitors, and bispecific T-cell engagers using a syndrome-based framework. Key topics include cytokine release syndrome and HLH/MAS, immune effector cell-associated neurotoxicity syndrome, respiratory failure related to pneumonitis and infection, cardiovascular emergencies such as myocarditis and arrhythmias, and other less common but ICU-relevant organ toxicities. We also summarize the major diagnostic conflicts in the ICU and propose general principles for organ support, immunomodulatory therapy, infection management, and multidisciplinary care.

**Conclusion:**

Immunotherapy-related critical illness is increasingly relevant in modern oncologic intensive care. Because these syndromes are often clinically overlapping yet potentially reversible, optimal management requires early ICU escalation, parallel diagnostic reasoning, and integrated supportive and syndrome-directed treatment.

## Introduction

In recent years, immunotherapy has fundamentally reshaped the treatment landscape of multiple malignancies and has delivered durable disease control for a subset of patients who previously had very limited therapeutic options (1–3). At the same time, its rapid expansion has introduced a spectrum of life-threatening complications that differs substantially from the traditional critical illness patterns seen in oncology (2, 4, 5). Whereas ICU admission in patients with cancer was historically driven mainly by severe infection, tumor progression, or toxicity from cytotoxic therapy, the immunotherapy era has brought a growing number of patients to the ICU because of treatment-induced hyperinflammation, organ-specific immune injury, or secondary infectious complications (2, 6, 7). These events may evolve rapidly into shock, respiratory failure, severe neurologic dysfunction, and multiorgan failure, thereby requiring timely intensive care support (5, 8–10).

The critical care implications of immunotherapy are further amplified by the heterogeneity of currently used treatment platforms (2, 3, 11). Chimeric antigen receptor T-cell (CAR-T) therapy, immune checkpoint inhibitors (ICIs), and bispecific T-cell engagers (BiTEs) all enhance antitumor immunity, but they do so through distinct mechanisms and therefore generate different patterns of critical illness (2, 11, 12). CAR-T cells and BiTEs are more commonly associated with early hyperinflammatory toxicity, most notably cytokine release syndrome (CRS), immune effector cell-associated neurotoxicity syndrome (ICANS), and infectious complications (8, 9, 13–15). In contrast, ICIs more often produce delayed and organ-specific immune-related adverse events (irAEs), including pneumonitis, myocarditis, encephalitis, and other severe inflammatory syndromes that may be uncommon overall but carry substantial ICU relevance and short-term mortality (1, 4, 10, 16, 17). As indications broaden and exposure increases, these complications are no longer confined to highly specialized centers, but are becoming an increasingly important part of contemporary oncologic critical care (2, 3, 11).

For intensivists, the principal challenge is not simply recognizing that such toxicities exist. Rather, it is the fact that their clinical presentation frequently overlaps with sepsis, opportunistic infection, tumor progression, drug-related organ dysfunction, and other conventional ICU syndromes (4, 5, 10, 14, 16). Fever, hypotension, hypoxemia, altered mental status, or troponin elevation may each reflect immune toxicity, infection, or both (5, 8, 10, 17). In many patients, these processes are not mutually exclusive but coexist and amplify one another (10, 16, 18). As a result, ICU decision-making in the immunotherapy era rarely follows a linear diagnostic pathway. Instead, it requires parallel evaluation, serial reassessment, early organ support, and timely consideration of immunomodulatory therapy under substantial uncertainty (2, 4, 10). Delayed recognition and delayed intervention may both adversely affect short-term outcomes (8, 10, 16).

Importantly, immunotherapy-related critical illness differs from many traditional oncology ICU scenarios in one essential respect: a substantial proportion of these emergencies represent treatment-related and potentially reversible complications rather than the inevitable manifestation of end-stage cancer (1, 5, 19). This distinction matters because it challenges the historical assumption that ICU admission in patients with advanced malignancy necessarily implies limited benefit from aggressive support (1, 7, 19). Increasing clinical experience suggests that with standardized pathways, modern organ support, and close multidisciplinary collaboration, a meaningful proportion of patients can survive the acute toxic phase and still derive oncologic benefit from immunotherapy (2, 6, 7, 19). Reassessing ICU triage, diagnostic reasoning, and treatment priorities in this setting is therefore both clinically necessary and conceptually important (2, 3, 10, 19).

This review does not aim to summarize all immune-related adverse events. Instead, it focuses on complications that are most relevant to ICU admission, organ support, and short-term mortality (2, 10). Using an ICU-centered and syndrome-based framework, we discuss hyperinflammatory syndromes, neurocritical complications, respiratory failure, cardiovascular emergencies, and other major organ toxicities, with particular emphasis on recognition, differential diagnosis, organ support, and immunomodulatory management (5, 10, 16, 17). We also highlight heterogeneity across treatment platforms and patient populations, and summarize current knowledge gaps regarding outcomes, rechallenge, and future research priorities (2, 3, 10, 19).

## Literature search strategy

This narrative review was informed by a structured literature search of PubMed, Embase, and Web of Science for English-language studies addressing severe toxicities associated with contemporary cancer immunotherapies, with particular emphasis on ICU-relevant complications related to CAR-T therapy, BiTEs, and ICIs. Priority was given to clinical guidelines, consensus statements, cohort studies, and key translational or mechanistic reports relevant to recognition, differential diagnosis, and critical care management. Additional references were identified through citation tracking of major reviews and primary studies.

### How immunotherapy has changed the landscape of critical illness in cancer patients

The widespread adoption of immunotherapy has altered not only oncologic outcomes, but also the clinical meaning of critical illness in patients with cancer (2, 3, 5). Historically, ICU admission in oncology was most often prompted by uncontrolled infection, tumor progression, postoperative complications, or the toxic effects of conventional chemotherapy (3, 19). In the immunotherapy era, however, an increasing proportion of critically ill patients are admitted because of treatment-triggered hyperinflammation, organ-specific immune injury, or secondary complications arising during toxicity management (2, 4, 16, 20). This shift is clinically important because it means that ICU physicians are no longer dealing exclusively with irreversible end-stage deterioration. In many cases, they are managing acute, treatment-related syndromes that may be severe but remain potentially reversible if recognized early and supported appropriately (6–8, 17, 20).

This new ICU landscape is shaped to a large extent by the immunotherapeutic platform used (2, 3, 19). CAR-T therapy, ICIs, and BiTEs all mobilize antitumor immunity, but they do so in biologically distinct ways and therefore generate different toxicity timelines, organ patterns, and critical care needs (2, 11, 12, 21). For intensivists, identifying the treatment platform is often one of the most informative early steps in clinical reasoning (2, 10, 15). In a patient presenting with fever, shock, hypoxemia, encephalopathy, or cardiac instability after immunotherapy, knowing whether the underlying exposure was CAR-T, ICI, or BiTE may be more helpful at the bedside than starting with an undifferentiated list of possible toxicities (2, 11, 15, 21).

### CAR-T therapy and other immune effector cell therapies

Among currently used immunotherapy platforms, CAR-T therapy has perhaps most clearly transformed the ICU phenotype of patients with hematologic malignancies (3, 11, 15). In addition to its remarkable antitumor activity in relapsed or refractory leukemia and lymphoma, CAR-T therapy is characterized by a relatively high incidence of acute systemic inflammatory toxicity (8, 11, 15). From an ICU perspective, its most defining complications are CRS and ICANS, both of which typically occur early after infusion and may progress rapidly from mild constitutional symptoms to vasodilatory shock, respiratory failure, severe encephalopathy, seizures, and multiorgan dysfunction (8, 9, 13, 15). Because these complications cluster within a relatively predictable time window, they lend themselves to anticipatory monitoring and early ICU engagement (9, 11, 15).

This feature fundamentally distinguishes CAR-T-related critical illness from many traditional oncology ICU scenarios. Patients admitted with severe CRS or ICANS are not necessarily experiencing treatment failure or terminal oncologic decline (7, 8, 17). On the contrary, ICU admission often occurs precisely when the immune therapy is biologically most active (8, 11, 13). For that reason, the role of the ICU in CAR-T programs is frequently proactive rather than purely reactive (9, 11, 15). Early escalation of monitoring, standardized grading of CRS and ICANS, and low-threshold organ support are often central to preserving reversibility and improving outcomes (8, 13, 15). In this setting, intensive care becomes part of the therapeutic pathway rather than merely a rescue destination (8, 15).

At the same time, CAR-T-related ICU risk is not limited to hyperinflammation itself. These patients often enter treatment after multiple prior lines of therapy and typically undergo lymphodepleting chemotherapy before infusion (3, 11, 15). They may also develop prolonged B-cell aplasia, hypogammaglobulinemia, and subsequent exposure to high-dose corticosteroids or other immunosuppressive agents (3, 11, 15). As a result, infection remains a major determinant of critical illness in this population (3, 11, 15). Importantly, infection does not simply compete with CRS as an alternative diagnosis; it may coexist with it, mimic it, or amplify it (8, 14, 15). The ICU challenge in CAR-T recipients is therefore not a transition from an “infection era” to a purely “toxicity era,” but rather the emergence of a critical care environment in which hyperinflammation and severe infection are closely intertwined (8, 14, 15).

### Immune checkpoint inhibitors

ICIs have changed the ICU landscape in a different and, in many ways, more deceptive manner (4, 10, 19). Although most immune-related adverse events are mild to moderate, a minority of patients develop fulminant organ-specific toxicities with substantial short-term mortality (1, 17, 20). Among the most ICU-relevant are pneumonitis, myocarditis, encephalitis, and other severe inflammatory syndromes affecting the lung, heart, nervous system, or gastrointestinal tract (4, 17, 22). Unlike CAR-T-associated toxicity, these events do not usually present as a clustered early systemic syndrome. Instead, they are often delayed, heterogeneous, and clinically disguised as more conventional causes of organ failure (16, 20, 22).

### Sustained diagnostic vigilance after ICI exposure

This delayed and organ-specific pattern has major implications for critical care practice. Unlike CAR-T- or BiTE-associated toxicity, which often occurs within a relatively predictable early window, severe ICI-related irAEs may develop weeks to months after treatment initiation and, in some cases, after treatment discontinuation (20, 22–24). Therefore, ICU clinicians should not exclude ICI toxicity solely because the most recent dose was not recent (1, 10, 20, 23). Any patient with prior exposure to PD-1, PD-L1, or CTLA-4 inhibition who presents with otherwise unexplained respiratory failure, myocardial injury, conduction abnormality, neurologic deterioration, severe colitis, hepatitis, nephritis, endocrine crisis, or systemic inflammatory dysfunction should be evaluated with irAEs in mind (20, 22–24). This sustained diagnostic vigilance is especially important because ICI-related critical illness often presents as organ-specific failure that mimics infection, tumor progression, comorbidity-related decompensation, or conventional ICU syndromes (16, 20, 23, 25).

The host background of ICI-treated patients also contributes to the critical care phenotype (6, 7, 16). Many such patients have solid tumors, are older, and carry substantial cardiopulmonary comorbidity, prior radiation exposure, or reduced organ reserve (20). As a result, once an irAE occurs, it may precipitate decompensation more rapidly than would be expected in a more resilient host (10, 16, 17). For example, a patient with lung cancer and limited pulmonary reserve may progress quickly from moderate checkpoint inhibitor pneumonitis to severe hypoxemic respiratory failure (10, 16), while a patient with underlying cardiovascular disease may deteriorate rapidly when ICI-associated myocarditis develops (10, 17). Thus, the ICU burden of ICI toxicity reflects not only the inflammatory event itself, but also the organ on which it lands (10, 16, 17, 22).

### Bispecific T-cell engagers and emerging immunotherapy platforms

BiTEs occupy an intermediate position between CAR-T therapy and ICIs in terms of critical care phenotype (3, 11, 12, 21). Like CAR-T cells, they directly activate T cells and are therefore associated with early inflammatory toxicity, most notably CRS and, less commonly, neurotoxicity including ICANS-like presentations (8, 12, 21, 26). From an ICU perspective, clinically significant BiTE-associated toxicity may present with fever, hypotension, hypoxemia, encephalopathy, seizure, capillary leak, infection, tumor lysis, or multiorgan dysfunction (21, 26–28). Although many events are low grade, severe cases may require ICU monitoring, vasopressors, respiratory support, or treatment interruption (21, 26–28).

BiTE-associated CRS shares several bedside features with CAR-T-associated CRS, including fever, vasodilation, oxygen requirement, and inflammatory marker elevation (12, 21, 26). However, the treatment context differs. CAR-T toxicity usually follows a one-time cellular infusion and is closely related to *in vivo* immune effector cell expansion, whereas BiTE toxicity often occurs during step-up dosing, early cycles, or dose escalation and may recur with subsequent doses (11, 12, 21, 26). This repeated dosing structure may allow dose interruption, delay, or modification when clinically significant toxicity develops (12, 21, 26). Thus, BiTE-related critical illness may be more pharmacologically modifiable than CAR-T-related toxicity, although severe CRS, neurotoxicity, or infection still requires rapid ICU assessment and organ support (21, 26, 29, 30).

The rapid expansion of BiTEs and other bispecific antibodies across hematologic malignancies, and increasingly into selected solid tumor settings, means that ICU teams are likely to encounter these complications more often (21, 26–28). However, dedicated real-world ICU outcome data for BiTE-associated critical illness remain limited compared with CAR-T therapy (21, 26–28). Current ICU management is therefore largely extrapolated from immune effector cell toxicity principles: early recognition of CRS and neurotoxicity, parallel evaluation for infection and tumor lysis, timely organ support, CRS-directed therapy such as tocilizumab and corticosteroids when indicated, treatment interruption when appropriate, and close multidisciplinary reassessment (26, 29–31).

### From treatment type to ICU prediction

The most important shift brought about by immunotherapy may therefore be conceptual rather than purely descriptive. What has changed is not only the list of complications, but also the starting point of ICU reasoning (2, 10, 19). In the past, the initial question in a critically ill patient with cancer was often whether the presentation reflected infection, tumor progression, or treatment-related toxicity in a broad sense. In the immunotherapy era, a more informative early question is often: what treatment platform was used, and what type of critical illness does that platform most commonly produce (10, 11, 15, 21)? For CAR-T and BiTE recipients with early fever, hypotension, hypoxemia, or neurologic change, hyperinflammatory syndromes and their overlap with infection should move immediately to the foreground (12, 14, 21, 26). For ICI-exposed patients with abrupt deterioration in a specific organ system, severe irAEs should remain high in the differential even when infection or preexisting comorbidity appears plausible (16–18, 20, 22).

**Figure 1:**
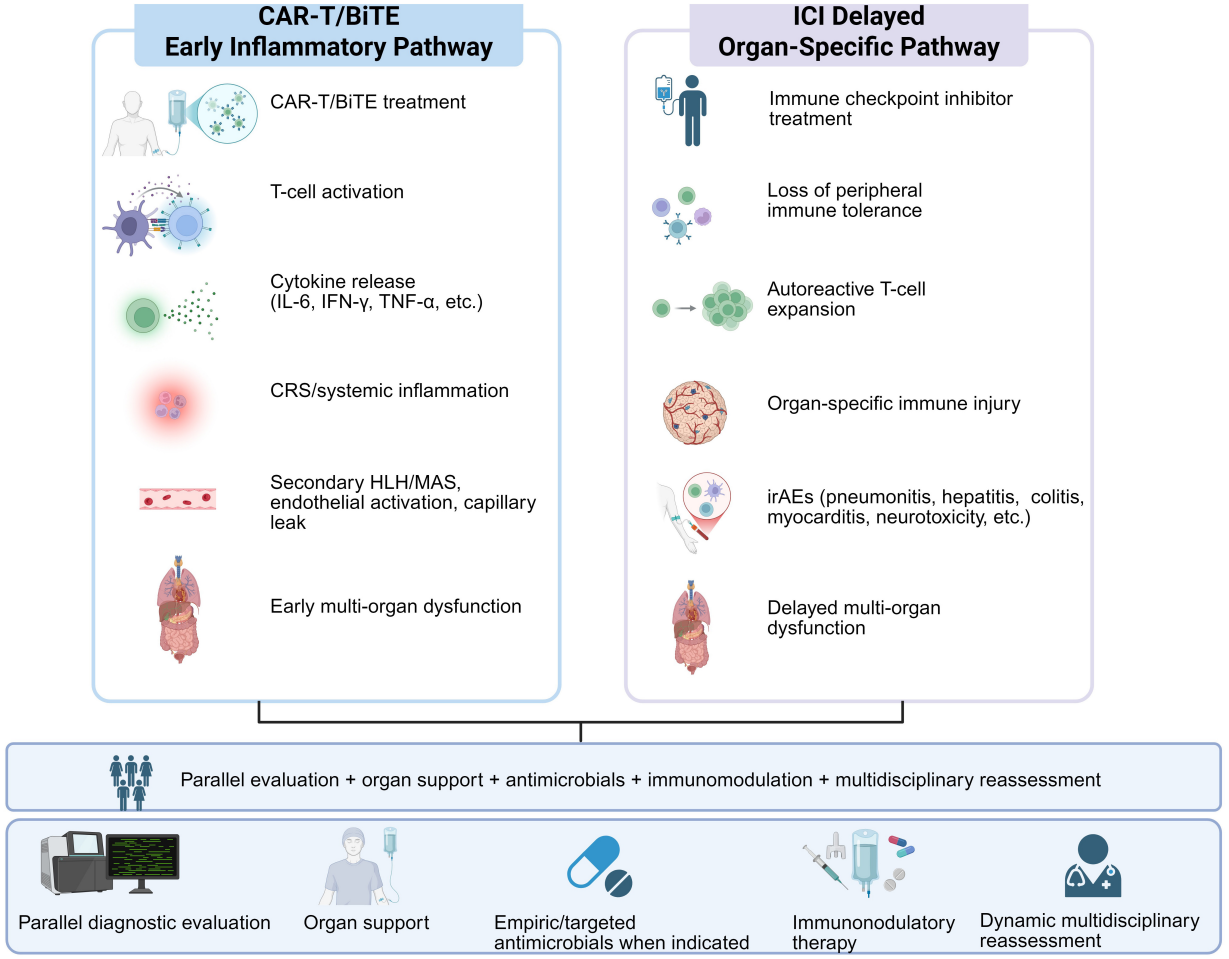
Schematic overview of major immunotherapy platforms, their principal toxicity patterns, ICU-relevant severe clinical manifestations, key diagnostic overlaps, and overarching ICU management priorities. CAR-T therapy, bispecific T-cell engagers, and immune checkpoint inhibitors are linked to immune effector-related toxicities, organ-specific immune toxicities, infection/sepsis/tumor progression overlap, and ICU priorities including early escalation, parallel diagnostic evaluation, organ support, immunomodulatory therapy, and multidisciplinary infection surveillance.

For this reason, the remainder of this review does not treat all immune toxicities as equally relevant to the ICU. Instead, it focuses on those clinical syndromes most closely linked to ICU admission, organ support, and short-term mortality (2, 10). This syndrome-based approach better reflects the practical transformation of oncologic critical care in the immunotherapy era: the ICU is no longer engaged only at the end of the cancer trajectory, but increasingly at the intersection of high-risk, treatment-related, and potentially reversible critical illness (17, 20). Table 1 summarizes the ICU-relevant patterns of critical illness associated with major immunotherapy platforms, including typical timing, dominant ICU syndromes, common competing diagnoses, key diagnostic tests, escalation red flags, first-line syndrome-directed therapy, organ support needs, and distinctive ICU considerations. Taken together, these platform-specific differences provide a practical bridge between treatment exposure and syndrome-based ICU management (15, 21). Figure 1 summarizes the major immunotherapy platforms, their typical toxicity timelines, dominant ICU syndromes, key diagnostic overlaps, ICU-relevant severe manifestations, and overarching management priorities, thereby introducing the syndrome-focused sections that follow.

**Table 1:** ICU-relevant patterns of critical illness across major immunotherapy platforms.

Therapeutic platform	Typical timing of severe toxicity	Dominant ICU syndromes	Key competing diagnoses/overlapping processes	Key diagnostic tests and escalation red flags	First-line syndrome-directed therapy	Common ICU organ support needs	Distinctive ICU considerations
CAR-T/immune effector cell therapies	Usually early, most often within days to 2 weeks after infusion; CRS often precedes or overlaps with neurotoxicity.	CRS; ICANS; infection; less commonly HLH/MAS; capillary leak and multiorgan dysfunction.	Sepsis; neutropenic infection; tumor lysis syndrome; drug-related encephalopathy; CNS infection; stroke; metabolic encephalopathy.	CRS grading; ICE score or structured neurologic assessment; CBC with differential; CRP; procalcitonin; ferritin trend; fibrinogen; triglycerides; coagulation profile; liver and renal function; lactate; blood, urine, respiratory, and catheter cultures; chest imaging; EEG, brain imaging, and CSF studies when neurologically indicated and feasible. Escalation red flags include vasopressor requirement, rapidly increasing oxygen need, declining ICE score, seizure, reduced consciousness, rapidly rising ferritin, hypofibrinogenemia, worsening cytopenias, progressive hepatic dysfunction, acute kidney injury requiring renal replacement therapy, or multiorgan dysfunction despite initial treatment.	Tocilizumab for clinically significant CRS; corticosteroids for severe or refractory CRS and ICANS; escalation to broader immunomodulation for refractory hyperinflammation or HLH/MAS according to multidisciplinary assessment.	Vasopressors; oxygen therapy or mechanical ventilation; airway protection; EEG-guided neurocritical care; seizure management; renal replacement therapy.	ICU deterioration may occur during the period of maximal antitumor activity; early ICU engagement, structured CRS/ICANS monitoring, infection surveillance, and repeated reassessment are especially important.
Bispecific T-cell engagers (BiTEs)	Usually early, especially during step-up dosing, ramp-up dosing, early treatment cycles, or after dose escalation; toxicity may recur with subsequent doses.	CRS; neurotoxicity including ICANS-like presentations; infection; fever; hypotension; hypoxemia; occasional capillary leak, tumor lysis, or multiorgan dysfunction.	Sepsis; infusion-related reaction; neutropenic infection; CNS infection; tumor lysis syndrome; disease progression; drug-related fever; metabolic encephalopathy.	Vital sign trajectory during and after dosing; CRS grading; neurologic assessment or ICE score when applicable; CBC with differential; CRP; procalcitonin; ferritin; renal function; electrolytes; uric acid and other tumor lysis markers; liver function; coagulation profile; microbiologic cultures; chest imaging when respiratory symptoms are present. Escalation red flags include persistent or recurrent fever with hypotension, oxygen requirement, vasopressor need, neurologic change, seizure, clinically significant capillary leak, tumor lysis, rising creatinine, or suspected infection during neutropenia or immunosuppression.	Temporary treatment interruption or dose delay when appropriate; corticosteroids and CRS-directed therapy, including tocilizumab when clinically indicated; tumor lysis prevention and treatment; empiric antimicrobials when infection cannot be excluded.	Hemodynamic support; oxygen therapy; neurologic monitoring; occasional ventilatory support; renal support when tumor lysis or multiorgan dysfunction develops.	Toxicity may resemble immune effector cell therapy, but repeated dosing and step-up schedules may allow earlier modification, interruption, or delay of treatment; ICU teams should be familiar with product-specific monitoring windows and escalation criteria.
Immune checkpoint inhibitors (ICIs)	Usually delayed; often weeks to months after treatment initiation, but may occur later or after discontinuation.	Pneumonitis; myocarditis; encephalitis or other neurologic irAEs; myocarditis-myositis-myasthenia overlap syndrome; severe colitis; hepatitis; nephritis; endocrine crisis.	Infection; tumor progression; radiation pneumonitis; pulmonary embolism; acute coronary syndrome; septic cardiomyopathy; stress cardiomyopathy; metabolic encephalopathy; stroke; infectious encephalitis; drug toxicity; ischemic organ injury.	Organ-directed testing according to presentation: chest CT, microbiologic studies, viral/fungal/Pneumocystis testing, bronchoscopy and bronchoalveolar lavage when safe; ECG, serial troponin, natriuretic peptides, echocardiography, and cardiac MRI when feasible; focused neurologic examination, brain CT or MRI, EEG, and CSF studies when feasible; liver tests, renal function, urinalysis, stool studies, glucose, ketones, cortisol, thyroid function, and electrolytes as indicated. Escalation red flags include rapidly worsening hypoxemia, ARDS, dynamic troponin elevation, new conduction abnormality, ventricular arrhythmia, syncope, cardiogenic shock, bulbar weakness, respiratory muscle weakness, seizure, reduced consciousness, severe colitis with bleeding, perforation or shock, acute liver failure, adrenal crisis, diabetic ketoacidosis, or progressive multiorgan dysfunction.	Hold suspected ICI in severe toxicity; corticosteroids are the main first-line treatment for most severe irAEs; additional organ-specific immunosuppression in corticosteroid-refractory cases; empiric antimicrobials when infection risk is substantial.	Oxygen therapy or mechanical ventilation; hemodynamic monitoring; vasopressors or inotropes; pacing or mechanical circulatory support in selected myocarditis cases; renal replacement therapy; neurocritical support; metabolic stabilization for endocrine crisis.	ICU recognition depends on sustained diagnostic vigilance rather than a predictable early toxicity window; organ-specific failure may be the dominant presentation, and severe irAEs should remain in the differential even when ICI exposure was remote.
Other emerging immunotherapy platforms	Variable and platform-dependent; may combine early inflammatory toxicity with delayed organ-specific injury depending on treatment mechanism and sequence.	Mixed phenotypes, including hyperinflammation, organ-specific toxicity, infection, capillary leak, respiratory failure, cardiovascular instability, neurologic dysfunction, and multiorgan failure.	Sepsis; opportunistic infection; treatment-related organ dysfunction; progression of malignancy; prior radiation injury; chemotherapy-related toxicity; comorbidity-related decompensation.	Detailed treatment timeline; platform-specific toxicity review; serial inflammatory markers; microbiologic studies; organ-directed imaging and laboratory testing; multidisciplinary review of current and prior anticancer therapies. Escalation red flags include rapidly progressive organ failure, simultaneous shock and hypoxemia, neurologic decline, dynamic myocardial injury, rising ferritin with cytopenias or coagulopathy, or persistent deterioration despite initial antimicrobial or immunomodulatory therapy.	Platform-specific when available; otherwise often extrapolated from CAR-T-, BiTE-, or ICI-based toxicity management principles; early empiric antimicrobials and immunomodulation should be balanced according to the dominant syndrome and infectious risk.	Depends on the dominant syndrome; may include vasopressors, oxygen therapy, mechanical ventilation, renal replacement therapy, neurocritical care, or advanced cardiovascular support.	ICU phenotypes may not fit neatly into existing categories; treatment mechanism, timing, host vulnerability, and multidisciplinary interpretation are essential for bedside decision-making.

Timing, severity, and dominant ICU manifestations may vary according to treatment construct, disease burden, prior therapies, host vulnerability, and local toxicity management practice. Immune toxicity, infection, tumor progression, and conventional critical illness may coexist; therefore, diagnostic evaluation and treatment often need to proceed in parallel rather than sequentially. CAR-T, chimeric antigen receptor T-cell; CRS, cytokine release syndrome; ICANS, immune effector cell-associated neurotoxicity syndrome; HLH/MAS, hemophagocytic lymphohistiocytosis/macrophage activation syndrome; ICE, immune effector cell-associated encephalopathy; CNS, central nervous system; CSF, cerebrospinal fluid; EEG, electroencephalography; ICI, immune checkpoint inhibitor; irAE, immune-related adverse event; ARDS, acute respiratory distress syndrome.

### Major ICU syndromes

#### Hyperinflammatory syndromes: CRS and HLH/MAS

Hyperinflammatory syndromes represent one of the most characteristic forms of immunotherapy-related critical illness and remain among the most common reasons for ICU admission after immune effector cell therapies (32–34). They are seen most often after CAR-T and BiTE exposure, although similar immune dysregulation may occasionally occur in other immunotherapeutic settings (21, 26, 35). From an ICU perspective, their importance lies not only in their frequency, but also in their tempo: patients may deteriorate within hours from persistent fever and constitutional symptoms to vasodilatory shock, hypoxemic respiratory failure, and multiorgan dysfunction (34–36). In this setting, the central challenge is rarely the recognition of inflammation alone, but rather determining whether a clinically worsening inflammatory state remains potentially controllable or is progressing toward fulminant immune dysregulation (2, 8, 11, 37).

Cytokine release syndrome (CRS) and hemophagocytic lymphohistiocytosis/macrophage activation syndrome (HLH/MAS) are best understood in critical care as overlapping hyperinflammatory phenotypes rather than completely discrete entities (11, 34, 37). CRS is more common and typically occurs earlier, particularly after CAR-T therapy, reflecting rapid activation of immune effector cells and downstream myeloid amplification (15, 34, 36). HLH/MAS, by contrast, usually represents a more severe and destabilized inflammatory state, characterized by progressive cytopenias, marked hyperferritinemia, coagulopathy, hepatic dysfunction, and escalating multiorgan failure (2, 11, 37). Although formal diagnostic frameworks distinguish the two syndromes, the more clinically relevant ICU question is often whether the patient is progressing from a broadly recognizable CRS-like presentation toward macrophage-dominant immune failure with increasing biologic and organ-level instability (2, 8, 37).

CRS remains the archetypal acute hyperinflammatory complication of immune effector cell therapy (15, 34, 36). In its early stages, it may present with fever, malaise, tachycardia, and mild oxygen requirement (8, 15, 36). In more severe forms, however, it may rapidly evolve into distributive shock, capillary leak, progressive hypoxemia, and multiorgan dysfunction requiring vasopressor support, mechanical ventilation, or renal replacement therapy (32–34). The clinical importance of CRS in the ICU is therefore not merely that it is common, but that it frequently unfolds within a relatively predictable treatment window and can worsen quickly enough to justify low-threshold escalation of monitoring and support (11, 15, 34). Unlike many conventional oncology ICU scenarios, severe CRS often occurs when anticancer therapy is biologically active rather than when disease control has failed, which is one reason why aggressive supportive care is often justified (15, 32, 34).

HLH/MAS carries a different prognostic and therapeutic weight. While less frequent than CRS, it is generally associated with greater biologic dysregulation and worse short-term outcomes (11, 33, 37). In the ICU, suspicion should rise when persistent fever and hemodynamic instability are accompanied by rapidly increasing ferritin, worsening cytopenias, deteriorating liver function, hypofibrinogenemia, and progressive organ failure despite initial CRS-directed management (2, 37). Importantly, HLH/MAS in this setting may emerge as a continuation of severe CRS, as a partially overlapping syndrome, or as a less common but highly destructive immune complication in other immunotherapy contexts (2, 11, 37). For intensivists, this distinction matters because the transition from CRS to HLH-like physiology often signals the need for more urgent escalation in both organ support and immunomodulatory intensity (2, 37).

The greatest diagnostic difficulty in hyperinflammatory syndromes is their overlap with sepsis (8, 14, 34, 38). Fever, shock, rising inflammatory markers, and organ dysfunction are shared features, and in many patients the two processes coexist rather than compete (8, 14, 38). Immune effector cell therapy recipients often have multiple infection-promoting exposures, including lymphodepleting chemotherapy, neutropenia, mucosal injury, central venous access, prolonged hospitalization, corticosteroid use, and additional immunosuppressive agents (3, 11, 15, 36). At the same time, infection itself may amplify preexisting inflammatory toxicity (8, 14, 38). For this reason, ICU management should not be based on the false premise that the patient must have either CRS or sepsis. More often, the practical task is to determine which process is dominant, whether both require immediate treatment, and how the balance changes over time (8, 14, 38).

No single biomarker is sufficient to reliably distinguish CRS, HLH/MAS, and infection at the bedside (14, 16, 38). Nevertheless, biomarkers remain useful for risk stratification and serial reassessment when interpreted as patterns rather than isolated values (14, 38–40). CRP may reflect IL-6-driven inflammation but is not specific for CRS, whereas procalcitonin may support bacterial infection when markedly elevated or dynamically rising, although it may also increase during severe systemic inflammation, shock, renal dysfunction, or cytokine-mediated injury (14, 38, 40). Ferritin is particularly helpful when interpreted dynamically: rapidly rising ferritin accompanied by worsening cytopenias, hypofibrinogenemia, hypertriglyceridemia, hepatic dysfunction, coagulopathy, or progressive organ failure should raise concern for HLH/MAS-like hyperinflammation rather than uncomplicated CRS alone (14, 37, 38, 40).

Emerging biomarkers may further support risk assessment but should not be treated as stand-alone discriminators (37, 38, 40, 41). Cytokine panels including IL-6, IL-10, IFN-γ, TNF-α, and granulocyte-macrophage colony-stimulating factor may provide insight into CRS severity, endothelial activation, and macrophage-dominant inflammation (14, 37, 40). Soluble interleukin-2 receptor, also known as soluble CD25, may support T-cell activation and HLH/MAS-like physiology when interpreted with ferritin kinetics, cytopenias, fibrinogen, triglycerides, and liver dysfunction (37, 40). However, these assays are not universally available in real time, thresholds are not standardized across platforms, and specificity is limited in critically ill patients (14, 37, 38, 40). Therefore, biomarker trends should guide reassessment and escalation, but should not delay organ support, antimicrobial therapy, or time-sensitive immunomodulation (14, 37, 38, 40).

Management of hyperinflammatory syndromes must integrate organ support and immune modulation from the outset (11, 15, 34). Standard ICU care remains essential, including hemodynamic support, respiratory support, renal replacement therapy when indicated, and correction of metabolic and coagulation abnormalities (32–34). At the same time, syndrome-directed therapy is often time-sensitive. Tocilizumab and corticosteroids remain central to the treatment of severe CRS (15, 34–36), while patients with suspected HLH/MAS or refractory inflammatory deterioration may require escalation to broader immunosuppressive strategies such as anakinra, etoposide, or other rescue approaches depending on local practice and multidisciplinary input (2, 37). In the ICU, the most consequential error is often not overtreatment, but delayed treatment in a patient whose inflammatory trajectory is clearly worsening (8, 15, 34, 37).

Overall, outcomes in hyperinflammatory syndromes have improved with earlier recognition, structured grading systems, and more consistent use of targeted anti-inflammatory therapy (8, 11, 15, 34). However, this improvement is not uniform. Patients with isolated or promptly controlled CRS often recover, whereas those who progress to HLH/MAS, refractory shock, or severe multiorgan failure remain at substantially higher risk (8, 11, 33, 37). From a critical care perspective, the key issue is therefore not whether inflammation is present, but whether the patient is crossing from a potentially reversible inflammatory syndrome into a progressively destabilizing immune catastrophe. That transition, rather than any single diagnostic term, is what should most strongly drive escalation in ICU management (2, 8, 37).

#### Neurocritical complications: ICANS and related encephalopathy

Neurocritical complications are among the most distinctive and challenging manifestations of immunotherapy-related critical illness, with immune effector cell-associated neurotoxicity syndrome (ICANS) representing the prototypical ICU-relevant phenotype (9, 15, 42, 43). Unlike shock or respiratory failure, neurologic toxicity may initially emerge through subtle abnormalities in language, attention, behavior, or orientation rather than overt physiologic collapse (9, 42, 43). Yet these early changes may rapidly progress to seizures, profound encephalopathy, loss of airway protection, or catastrophic cerebral edema. For intensivists, the major importance of ICANS lies in this mismatch between early presentation and later severity: what initially appears to be mild cognitive dysfunction may in fact represent the beginning of a time-sensitive neurocritical syndrome (9, 42, 43).

ICANS is generally understood as a central nervous system manifestation of broader immune dysregulation rather than a completely isolated neurologic event (9, 15, 42, 43). It often develops in close temporal association with CRS, although it may follow it, overlap with it, or occasionally emerge after systemic inflammatory signs have already begun to improve (9, 13, 42, 43). Mechanistically, endothelial activation, blood-brain barrier dysfunction, and cytokine-mediated neuroinflammation appear to play central roles (14, 16, 42, 43). From an ICU standpoint, however, mechanistic detail is less important than its clinical implication: neurologic deterioration may evolve even after the most obvious systemic inflammatory features have abated, and therefore requires continued vigilance beyond the peak of CRS (9, 42, 43).

The clinical spectrum of ICANS is broad. Early manifestations often include impaired attention, word-finding difficulty, disorganized speech, agraphia, slowed responses, or fluctuating confusion (9, 42, 43). As severity increases, patients may develop delirium, aphasia, tremor, reduced level of consciousness, seizures, or non-convulsive status epilepticus. In the most severe cases, cerebral edema and intracranial hypertension may develop, creating an immediately life-threatening neurologic emergency (9, 42, 43). The ICU relevance of ICANS is therefore not limited to diagnostic classification; it is defined by two practical questions: whether the patient is losing the ability to protect the airway, and whether occult seizure activity or impending cerebral edema is being missed.

Early neurologic assessment is particularly important because the first signs of ICANS may be more cognitive than dramatic. In patients recently exposed to CAR-T or other immune effector therapies, abrupt difficulty with naming, writing, attention, or orientation should not be dismissed as fatigue, anxiety, medication effect, or nonspecific delirium without further evaluation (9, 42, 43). These changes may represent the earliest clinically detectable phase of a deteriorating neuroinflammatory process. At the same time, the severity of ICANS does not always track perfectly with the current severity of CRS. Improvement in systemic inflammation should not be assumed to indicate neurologic safety, particularly in patients who have already demonstrated high-grade inflammatory toxicity (9, 13, 42, 43).

The central diagnostic challenge is that altered mental status in the ICU is intrinsically nonspecific. ICANS must be differentiated from infectious encephalitis, sepsis-associated encephalopathy, metabolic derangement, drug-related delirium, stroke, intracranial hemorrhage, and progression of underlying CNS malignancy or prior neurologic injury (9, 15, 42, 43). In practice, this is rarely a binary decision. The relevant question is not whether ICANS is theoretically possible, but whether it is sufficiently plausible and sufficiently dangerous to justify immediate neurocritical care action while alternative diagnoses are investigated in parallel (9, 10, 42). As in other immunotherapy-related ICU syndromes, diagnostic uncertainty should prompt structured parallel evaluation rather than therapeutic delay (9, 10, 42).

No single test definitively confirms ICANS in a critically ill patient. Neuroimaging may be normal, especially early in the course, and its greatest value often lies in excluding hemorrhage, infarction, or mass effect rather than proving immune-mediated neurotoxicity (9, 42, 43). EEG is particularly important because non-convulsive seizure activity may be clinically occult and yet highly relevant to management (9, 42, 43). Lumbar puncture may help exclude infectious meningoencephalitis and may provide supportive inflammatory data, but it should not be treated as a prerequisite for time-sensitive intervention in an unstable patient (9, 42). These tools are most useful when understood as components of a risk-stratifying neurocritical care pathway rather than as steps in a rigid sequential diagnostic algorithm.

Management of ICANS centers on timely recognition, airway protection, seizure prevention or treatment, and early anti-inflammatory therapy (9, 15, 42, 43). In contrast to CRS, where tocilizumab often plays a central role, neurotoxicity is more strongly associated with corticosteroid-based treatment strategies (8, 9, 42, 43). In patients with progressive cognitive decline, reduced consciousness, seizures, or signs of increasing intracranial pressure, delay in corticosteroid initiation may be more harmful than treatment based on an imperfect working diagnosis (9, 42, 43). For ICU patients, the practical priority is often not proving ICANS conclusively, but preventing secondary neurologic injury while the differential diagnosis remains open (9, 42).

Supportive care is equally critical. Patients with worsening encephalopathy may require early endotracheal intubation for airway protection and safe delivery of sedation, imaging, EEG monitoring, and intracranial pressure-directed therapy (9, 15, 42, 43). Seizure management, head-of-bed elevation, targeted osmotherapy when indicated, and close neurologic reassessment all remain central to ICU care (9, 42, 43). Importantly, ICANS rarely occurs in a vacuum. Many affected patients also carry systemic inflammatory burden, infection risk, or residual hemodynamic instability, which means neurocritical care must be integrated with broader ICU management rather than delivered as an isolated neurologic protocol (9, 15, 42).

Most patients with ICANS improve with early recognition and structured management, and many recover without major persistent deficits (9, 15, 42, 43). However, reversibility should not be mistaken for triviality. Severe cases may still be complicated by prolonged encephalopathy, status epilepticus, cerebral edema, infection during immunosuppressive treatment, and longer-term cognitive or functional impairment (9, 42, 43). From an ICU perspective, the key determinant of outcome is often not whether the syndrome can eventually be named, but whether the initial window for neurocritical intervention was recognized early enough to prevent irreversible deterioration. In that sense, ICANS exemplifies a broader principle in immunotherapy-related critical illness: the most important decisions are often made before the full diagnostic picture is available (9, 42).

#### Respiratory failure: pneumonitis, infection, and overlap syndromes

Respiratory failure is one of the most clinically consequential ICU presentations in the era of cancer immunotherapy (16, 20, 24, 44). Its importance lies not only in its frequency and potential severity, but also in the fact that it exemplifies the diagnostic uncertainty that defines many immunotherapy-related critical illnesses (10, 16, 20). In patients exposed to immune checkpoint inhibitors (ICIs), checkpoint inhibitor pneumonitis is among the most ICU-relevant organ-specific toxicities and may progress from mild inflammatory lung injury to severe hypoxemic respiratory failure or acute respiratory distress syndrome (ARDS) (16, 20, 24, 44). At the same time, infection remains a major driver of respiratory deterioration across multiple immunotherapy platforms, particularly in patients with prior chemotherapy, prolonged corticosteroid exposure, immune effector cell therapy, or additional immunosuppressive treatment (3, 15, 18, 28). From an ICU perspective, respiratory failure after immunotherapy is therefore rarely explained by a single process (14, 16, 18).

The defining challenge in this setting is not simply identifying pneumonitis, but determining whether immune-mediated lung injury, infection, tumor progression, or a combination of these processes is driving the clinical picture (20, 24, 28, 44). Checkpoint inhibitor pneumonitis is typically a noninfectious inflammatory injury of the pulmonary parenchyma, often occurring weeks to months after treatment initiation and sometimes even after treatment discontinuation (16, 20, 44). However, the same patient may also have bacterial pneumonia, opportunistic infection, radiation-related lung vulnerability, malignant lymphangitic spread, or hemorrhagic pulmonary complications (16, 18). These processes are not mutually exclusive. In many critically ill patients, infection acts not merely as an alternative diagnosis, but as a mimic, a trigger, or a coexisting complication that amplifies respiratory compromise (14, 16, 18).

This overlap is especially relevant in the ICU because early manifestations are often nonspecific. Dyspnea, cough, hypoxemia, new infiltrates, and inflammatory marker elevation may all be seen in immune-mediated pneumonitis, bacterial or fungal pneumonia, viral pneumonitis, or progressive thoracic malignancy. Imaging may narrow the differential but rarely closes it. Ground-glass opacities, organizing pneumonia-like patterns, multifocal consolidation, and diffuse interstitial changes may all be consistent with checkpoint inhibitor pneumonitis, yet none are sufficiently specific to exclude infection or tumor-related pathology (16, 44). Thus, the diagnostic task is not to identify a pathognomonic radiographic pattern, but to interpret imaging within the broader clinical and therapeutic context.

Treatment history and host background are often more informative than any single radiologic or laboratory feature (16, 20, 24, 44). Recent ICI exposure, duration of treatment, prior irAEs, ongoing corticosteroid or second-line immunosuppressive therapy, prior thoracic radiation, chronic lung disease, and the underlying malignancy all substantially alter the pretest probability of the competing diagnoses (16, 20, 44). For example, in a patient with lung cancer and poor baseline pulmonary reserve, even a moderate inflammatory insult may precipitate severe hypoxemia (10, 16). In contrast, in a profoundly immunosuppressed patient after CAR-T therapy, diffuse infiltrates may more strongly raise concern for opportunistic infection, even in the presence of systemic inflammation (15, 18, 28). The same respiratory syndrome does not carry the same meaning across different host backgrounds (16, 20).

In practice, the ICU question is often not whether pneumonitis is present, but whether it is the dominant driver of respiratory failure at that moment (10, 16, 20). This distinction matters because treatment delay in either direction may be costly. Waiting for complete diagnostic closure before initiating corticosteroids may allow fulminant immune-mediated lung injury to progress (10, 16). Conversely, prematurely treating all cases as pneumonitis without adequate infectious evaluation may expose the patient to escalating immunosuppression in the setting of unrecognized infection (14, 16, 18). High-quality ICU care therefore depends on structured parallel evaluation rather than a sequential “rule-out first, treat later” model.

Bronchoscopy and bronchoalveolar lavage can be highly informative in selected patients, particularly for identifying bacterial, fungal, viral, or Pneumocystis infection and for supporting a noninfectious inflammatory process when microbiologic data are unrevealing (14, 16, 18). However, in critically ill patients with severe hypoxemia or hemodynamic instability, these procedures may be poorly tolerated or logistically unsafe. Their value lies in reducing uncertainty and informing subsequent treatment refinement, not in serving as a mandatory prerequisite for initiating therapy (10, 16). In other words, invasive diagnostics should support ICU decision-making, not delay it.

Management typically requires concurrent escalation of respiratory support, microbiologic evaluation, empiric antimicrobial coverage, and consideration of immunomodulatory therapy (10, 16, 20). Standard critical care principles remain central, including high-flow oxygen, noninvasive or invasive ventilation when indicated, lung-protective ventilation, prone positioning, and, in carefully selected patients, extracorporeal support (10, 16). These interventions are not adjuncts to “real treatment”; they are often the measures that preserve reversibility while etiologic and anti-inflammatory therapies begin to work.

When the clinical suspicion for immune-mediated pneumonitis is high and respiratory failure is worsening, systemic corticosteroids are generally favored early rather than late (10, 16, 24, 44). This is particularly true in patients with compatible timing, imaging, and no immediately dominant alternative diagnosis (16, 44). Yet even in such cases, empiric antimicrobial therapy often remains appropriate, especially in ICU patients with substantial infectious risk or when severe hypoxemia leaves little margin for diagnostic delay (14, 16, 18). The most practical ICU approach is therefore not to choose between infection and pneumonitis at the outset, but to treat both possibilities proportionately while continuing to refine the differential diagnosis.

Outcomes in immunotherapy-associated respiratory failure vary substantially according to severity, underlying cause, and speed of intervention (16, 20, 24, 44). Many patients with moderate pneumonitis recover with corticosteroids and supportive care, but those who progress to ARDS, prolonged mechanical ventilation, or extracorporeal support face substantially higher mortality and morbidity (16, 20, 44). Importantly, hospital survival does not necessarily imply full pulmonary recovery. Persistent exertional intolerance, radiographic fibrosis, and reduced lung function may continue long after the acute illness has resolved (16, 44). From an ICU perspective, respiratory failure after immunotherapy therefore illustrates a central principle of this field: the most consequential decision is often not assigning a definitive label at presentation, but determining how to act safely and decisively while the dominant pathophysiology is still evolving.

#### Cardiovascular emergencies: myocarditis, arrhythmias, and shock

Cardiovascular emergencies are less common than hyperinflammatory syndromes or respiratory failure in patients receiving immunotherapy, but they carry disproportionate ICU relevance because deterioration may be abrupt, difficult to predict, and rapidly fatal (17, 20, 24, 45). Among these complications, immune checkpoint inhibitor-associated myocarditis is the most feared and clinically consequential (10, 17, 20, 45). Its significance does not stem from high incidence, but from the fact that even initially subtle presentations may quickly progress to conduction failure, malignant arrhythmia, severe ventricular dysfunction, or cardiogenic shock (10, 17, 45, 46). In the ICU, this is one of the clearest examples of a complication that is uncommon overall but too dangerous to miss.

ICI-associated myocarditis is generally understood as an immune-mediated attack on the myocardium and conduction system following loss of immune tolerance (10, 17). Although mechanistic detail continues to evolve, its clinical meaning is already clear: injury may extend beyond contractile myocardium to include the electrical system, making rhythm instability as important as pump failure (10, 17, 46). This helps explain why some patients deteriorate before a profound reduction in ejection fraction becomes apparent. From a critical care perspective, myocarditis should therefore not be conceptualized solely as an inflammatory cardiomyopathy. It is better understood as a time-sensitive cardiovascular toxicity that may compromise both mechanical and electrical cardiac stability.

The clinical spectrum is broad. Some patients present only with fatigue, chest discomfort, palpitations, or asymptomatic biomarker elevation, whereas others develop syncope, high-grade atrioventricular block, ventricular arrhythmias, acute heart failure, or frank cardiogenic shock (10, 17, 45, 46). The ICU relevance of myocarditis is not determined by how dramatic the initial symptoms are, but by how rapidly the syndrome can evolve once clinically apparent. A patient with modest troponin elevation and minor ECG changes may deteriorate far more quickly than a clinician accustomed to more conventional forms of myocardial injury might expect (10, 17). This dissociation between early presentation and potential severity is one reason why diagnostic delay is especially dangerous.

Another important feature is overlap with other immune-mediated syndromes. Myocarditis may coexist with myositis and myasthenia gravis-like syndromes, producing a highly unstable phenotype in which cardiac injury, skeletal muscle inflammation, bulbar dysfunction, and respiratory muscle weakness occur together (10, 46–48). These overlap syndromes are particularly important in the ICU because they increase complexity across multiple physiologic domains at once: patients may require cardiac monitoring and circulatory support while simultaneously facing airway compromise, aspiration risk, or ventilatory failure. The issue is not simply that more organs are involved, but that several life-sustaining systems may decompensate in parallel.

The major diagnostic challenge is that the earliest clues to myocarditis are often nonspecific. Troponin elevation, hypotension, ECG abnormalities, and lactate elevation are all common in the ICU and may also reflect acute coronary syndrome, sepsis-related myocardial dysfunction, pulmonary embolism, stress cardiomyopathy, or background structural heart disease (10, 17). What distinguishes myocarditis in this setting is not any single test result, but the need to assign it sufficient priority in the differential diagnosis when the treatment context makes it plausible. In patients with recent ICI exposure, new conduction abnormalities, unexplained myocardial injury, syncope, ventricular arrhythmia, or circulatory collapse should immediately raise concern for immune-mediated cardiac toxicity, even before definitive confirmation is available (10, 17, 20, 45).

Bedside assessment begins with continuous telemetry, serial ECGs, cardiac biomarkers, and echocardiography. These tools are especially valuable because they are rapidly available, repeatable, and directly relevant to ICU decision-making. ECG may reveal new conduction disease or diffuse repolarization abnormalities; biomarkers may demonstrate dynamic injury; echocardiography may identify impaired ventricular function, pericardial effusion, or hemodynamic compromise (10, 17). Cardiac magnetic resonance imaging can add diagnostic confidence by demonstrating inflammation and edema, but its role in critical care is primarily supportive rather than gatekeeping. In unstable patients, it should not delay high-priority interventions such as close monitoring, hemodynamic stabilization, escalation of rhythm management, or immunosuppressive therapy.

Management requires a combination of early immune modulation and aggressive cardiovascular support. In clinically high-risk presentations, prompt discontinuation of the suspected ICI and early initiation of high-dose corticosteroids are generally favored (10, 17, 20, 45). The rationale is not merely theoretical: once conduction failure, malignant arrhythmia, or progressive shock develops, the opportunity to prevent catastrophic deterioration narrows quickly (10, 17). As in other immunotherapy-related critical illnesses, the most consequential ICU error is often not overtreatment, but waiting for diagnostic certainty in a syndrome where the cost of delay may be substantial.

However, pharmacologic immunosuppression alone is rarely sufficient in the most severe cases. Many of the deaths associated with ICI-related myocarditis occur because the patient crosses a hemodynamic or electrophysiologic threshold before anti-inflammatory therapy has had time to work (10, 17, 45). For that reason, ICU management must also focus on what preserves immediate reversibility: continuous rhythm monitoring, early recognition of high-grade atrioventricular block, preparedness for temporary pacing, treatment of ventricular arrhythmias, vasopressor or inotropic support when needed, and, in selected patients, mechanical circulatory support or extracorporeal life support (10, 17, 46). In this syndrome, organ support is not secondary to treatment. It is the intervention that may keep the patient alive long enough for treatment to matter.

Escalation beyond corticosteroids may be required in refractory or fulminant cases, including intravenous immunoglobulin or other second-line immunosuppressive approaches depending on local practice and multidisciplinary expertise (10, 46, 47). However, the evidence base remains limited and is driven largely by retrospective series, case reports, and expert consensus (10, 17). This uncertainty reinforces an important ICU principle: although precise therapeutic sequencing remains incompletely defined, failure to recognize severity early is more dangerous than lack of a fully standardized rescue algorithm.

Outcomes remain guarded, particularly in patients with malignant arrhythmias, high-grade conduction block, overlap syndromes, or cardiogenic shock requiring advanced support (10, 17, 45, 46). Even among survivors, persistent ventricular dysfunction, conduction abnormalities, or the need for long-term cardiology follow-up may remain relevant. Rechallenge with the same immune checkpoint inhibitor after severe myocarditis is generally discouraged, especially in patients who required ICU care or advanced cardiovascular support (10, 17, 20). From an ICU perspective, the most important lesson is straightforward: cardiovascular toxicity after immunotherapy is rarely a diagnostic curiosity. It is a low-frequency, high-consequence emergency in which early suspicion, rapid monitoring, and decisive support often matter more than perfect diagnostic closure.

### Other organ toxicities with occasional ICU relevance

Although hyperinflammatory syndromes, neurotoxicity, respiratory failure, and cardiovascular emergencies account for most of the immediate ICU focus in immunotherapy-related critical illness, other organ toxicities also remain clinically relevant (1, 5, 23, 24). Hepatic, renal, endocrine, gastrointestinal, hematologic, and cutaneous complications are all well recognized in immuno-oncology practice (1, 10, 23). However, from an ICU perspective, these toxicities are less often the primary reason for admission than the syndromes discussed above. Their critical care importance more commonly lies in three contexts: they may occasionally present as fulminant organ failure, they may complicate the course of another dominant immune-related syndrome, or they may contribute to evolving multisystem dysfunction in a critically ill host (10, 20, 23, 24).

This distinction is important because the ICU relevance of these toxicities should not be judged by their outpatient frequency. Hepatitis and nephritis, for example, are not rare in routine oncologic care, but isolated moderate liver enzyme elevation or creatinine rise seldom drives ICU admission on its own (1, 10, 23). By contrast, the minority of patients who develop fulminant hepatic injury, severe renal failure requiring renal replacement therapy, endocrine crisis, bowel perforation, massive bleeding risk, or refractory metabolic derangement may enter the ICU through a very different pathway (10, 20, 23, 24). In these cases, the critical care question is not simply whether immune toxicity is present, but whether organ dysfunction is severe enough to threaten reversibility, and whether it reflects isolated organ injury or a broader process of systemic immune dysregulation.

Immune-related hepatic toxicity illustrates this principle well. In many patients, liver involvement presents as asymptomatic transaminase elevation or mixed hepatocellular-cholestatic injury that can be managed outside the ICU (1, 23). In critically ill patients, however, hepatic dysfunction often appears in a more complex context. It may reflect severe immune-mediated hepatitis, but it may also be part of CRS, HLH/MAS, sepsis-associated cholestasis, drug-induced liver injury, ischemic hepatopathy, or tumor infiltration (2, 8, 23, 37). No biochemical pattern is entirely specific in the ICU. What matters more is whether liver dysfunction is worsening rapidly, whether coagulopathy and encephalopathy are developing, and whether the patient is entering a trajectory of acute liver failure or multisystem collapse (10, 23). The practical ICU task is therefore syndromic and dynamic: to identify the minority of patients in whom hepatic injury is becoming clinically decisive while simultaneously maintaining broad etiologic vigilance.

Renal toxicity follows a similar pattern. Immune checkpoint inhibitor-associated nephritis may present insidiously and is often recognized through rising creatinine, sterile pyuria, or mild proteinuria (1, 10, 23). In contrast, acute kidney injury in the ICU after CAR-T or other immune effector therapies is frequently multifactorial, driven by hypotension, capillary leak, tumor lysis, nephrotoxic exposure, sepsis, and inflammatory organ dysfunction rather than isolated renal autoimmunity (15, 26, 49). For intensivists, the significance of renal injury lies less in assigning a precise nephrologic label at the bedside and more in recognizing what it signals physiologically: worsening perfusion failure, escalating multiorgan dysfunction, or the need for urgent renal replacement therapy (15, 49). In this context, treatment of kidney injury often begins with treatment of the systemic critical illness that produced it.

Other organ toxicities may also become ICU-relevant in selected cases. Endocrine complications such as immune-mediated diabetes may present as diabetic ketoacidosis (22, 24, 50, 51), while adrenal crisis or severe thyroid dysfunction may amplify circulatory instability and encephalopathy (10, 23, 52, 53). Gastrointestinal toxicities, including severe colitis, may lead to dehydration, sepsis, hemorrhage, or perforation (1, 10, 20, 23, 24). Hematologic toxicities may increase bleeding or infectious vulnerability (10, 15, 49), and severe cutaneous reactions may compromise barrier integrity and worsen critical illness through fluid loss or secondary infection (1, 10, 23). These complications are not the central focus of most ICU admissions after immunotherapy, but they should not be dismissed as peripheral. In the critically ill patient, they may serve as amplifiers of instability or as clues to broader multisystem immune involvement.

The management principles for these less common ICU-relevant toxicities are consistent with the broader framework of this review. Organ support must remain the immediate priority, while immunomodulatory therapy is considered according to the suspected syndrome, pace of deterioration, and competing diagnoses (2, 10, 23). At the same time, intensivists should avoid treating isolated laboratory abnormalities as if they were automatically equivalent to fulminant immune toxicity. The real challenge is to distinguish the many patients with clinically important but non-dominant organ injury from the smaller subset whose hepatic, renal, endocrine, gastrointestinal, or hematologic dysfunction is becoming a major determinant of short-term outcome (10, 20, 23, 24).

From a critical care standpoint, the importance of these toxicities lies precisely in their imbalance between frequency and ICU centrality. They are common enough to matter, but not common enough to define the field. Their role in the ICU is often contextual rather than primary: they help shape the severity, reversibility, and management complexity of immunotherapy-related critical illness, even when they are not the dominant syndrome at presentation.

### Diagnostic and differential challenges in the ICU

The diagnostic challenge in immunotherapy-related critical illness is rarely one of unfamiliarity alone. Most intensivists can learn the major names of immunotherapy-associated toxicities. What is substantially more difficult is that these syndromes almost never present in cleanly separated textbook forms (10, 16, 17, 23, 25). Patients arrive in the ICU with fever, shock, hypoxemia, altered mental status, arrhythmia, rising troponin, acute kidney injury, or multiorgan dysfunction - patterns that are deeply familiar in critical care but not specific to immune-mediated toxicity (2, 10, 15, 23, 31). The central problem is therefore determining whether a given presentation is primarily driven by immune toxicity, infection, tumor progression, conventional critical illness physiology, or some combination of these processes (10, 14, 16, 23, 31).

In practice, four principles help structure this process. First, treatment platform and time window matter: CAR-T and BiTE exposures favor early hyperinflammatory and neurotoxic syndromes, whereas ICIs more often generate delayed organ-specific toxicities (15, 20, 26, 31). Second, the dominant organ phenotype should shape the initial diagnostic frame (10, 16, 17, 23). Third, no single test is decisive in most ICU scenarios; biomarkers, imaging, and even invasive testing usually reduce uncertainty without eliminating it (23, 40, 54–57). Fourth, management often must begin before the diagnosis is fully resolved, so these patients are best served by parallel evaluation and treatment rather than a rigid sequential algorithm (25, 31, 57, 58).

### CRS vs. sepsis

Among all diagnostic conflicts in immunotherapy-related critical illness, the overlap between CRS and sepsis is one of the most clinically important (31, 39, 54). Both may present with fever, hypotension, vasodilation, capillary leak, elevated inflammatory markers, and progressive organ dysfunction, and in many patients after immune effector cell therapy the two processes coexist (15, 31, 38, 39). The practical ICU problem is therefore not choosing between CRS and sepsis as mutually exclusive categories, but determining which process is dominant at a given moment and which cannot safely go untreated.

Time course and treatment context are often more informative than any isolated biomarker. Early post-infusion fever and hemodynamic instability in a CAR-T recipient strongly support CRS, whereas focal infectious signs, profound neutropenia, recent steroid escalation, or hemodynamic deterioration out of proportion to the known toxicity trajectory heighten concern for infection (11, 15, 31, 32, 38). Ferritin, CRP, procalcitonin, cytokine profiles, and microbiologic studies may all help, but none reliably resolves the distinction on their own (14, 38, 39, 54). As a result, blood cultures, imaging, microbiologic sampling, hemodynamic support, empiric antimicrobials, and anti-inflammatory therapy often need to proceed in parallel (8, 14, 31, 38, 39).

### ICANS vs. infection, stroke, and metabolic encephalopathy

Altered mental status is among the least specific manifestations in the ICU, which makes neurotoxicity particularly difficult to diagnose. ICANS after CAR-T or related therapies must compete diagnostically with infectious encephalitis, sepsis-associated encephalopathy, metabolic derangement, medication-related delirium, ischemic stroke, intracranial hemorrhage, and progression of underlying CNS disease (9, 42, 43, 55, 59). The challenge is that the early manifestations of ICANS - language disturbance, inattention, disorientation, and cognitive slowing - may appear deceptively subtle, while the consequences of delayed recognition may be severe (9, 42, 43, 59).

Treatment context and timing matter: recent immune effector cell therapy, concurrent or recent CRS, and an early pattern of language or attention impairment support ICANS, whereas focal deficits, meningismus, severe metabolic disarray, or structural lesions may point more strongly toward competing diagnoses (9, 13, 42, 43, 59). Brain imaging, EEG, laboratory testing, and cerebrospinal fluid evaluation refine management and exclude dangerous alternatives, but in unstable patients airway protection, seizure management, and anti-inflammatory treatment may need to proceed before the diagnostic picture is complete (9, 42, 43, 55, 56, 59). No single test definitively confirms ICANS in a critically ill patient, and the value of MRI, lumbar puncture, and EEG often lies more in excluding dangerous alternatives and stratifying severity than in providing a binary confirmation.

### Pneumonitis vs. infection and tumor progression

Respiratory failure after immunotherapy often reflects several plausible pathophysiologic processes at once. ICI-associated pneumonitis may cause progressive hypoxemia, diffuse infiltrates, and ARDS-like physiology; infection may produce a nearly indistinguishable syndrome, especially in patients with prior corticosteroid exposure or other immunosuppression; and tumor progression, malignant lymphangitic spread, pulmonary hemorrhage, or prior radiation injury may further complicate the picture (16, 18, 20, 44, 60–62).

The most useful early information often comes from chronology and clinical context rather than any single imaging feature. Chest CT may support a working diagnosis, and bronchoscopy or bronchoalveolar lavage may be valuable when opportunistic infection is a realistic concern, but these are diagnostic aids rather than mandatory gates to treatment (16, 23, 44, 62, 63). In severely hypoxemic or unstable patients, respiratory support, microbiologic evaluation, empiric antimicrobial therapy, and corticosteroid treatment for suspected immune-mediated lung injury may all need to begin at the same time (10, 16, 23, 31, 62, 63). In this setting, the decisive ICU question is often not whether pneumonitis is present in principle, but whether it is the dominant driver of respiratory failure at that moment and whether delaying treatment in either direction would be more dangerous.

### Myocarditis vs. acute coronary syndrome and septic cardiomyopathy

ICI-associated myocarditis presents a different but equally important diagnostic conflict. Troponin elevation, ECG abnormalities, hypotension, and lactate elevation are all common in the ICU and may result from acute coronary syndrome, sepsis-related myocardial dysfunction, pulmonary embolism, stress cardiomyopathy, or structural heart disease (17, 23, 64, 65). What makes myocarditis particularly dangerous is that it may initially appear minor while rapidly progressing to conduction block, malignant arrhythmia, or cardiogenic shock (45, 58, 64, 65).

Recent exposure to PD-1, PD-L1, or CTLA-4 blockade, new conduction system abnormalities, unexplained myocardial injury, syncope, ventricular arrhythmia, or overlap features such as myositis or neuromuscular weakness should all raise concern (45–47, 64, 65). Telemetry, serial ECGs, biomarkers, and echocardiography form the first line of bedside assessment, while cardiac MRI may add diagnostic support when the patient is stable enough to undergo it (17, 58, 64, 65). In patients with strong clinical suspicion and evolving electrical or hemodynamic instability, monitoring, rhythm support, hemodynamic stabilization, and timely corticosteroid initiation should not wait for complete diagnostic closure (17, 23, 58, 64, 65). A multimodal diagnostic strategy may improve confidence, but in fulminant cases the role of diagnosis is to support urgent action rather than delay it.

### Diagnosing under uncertainty

**Figure 2:**
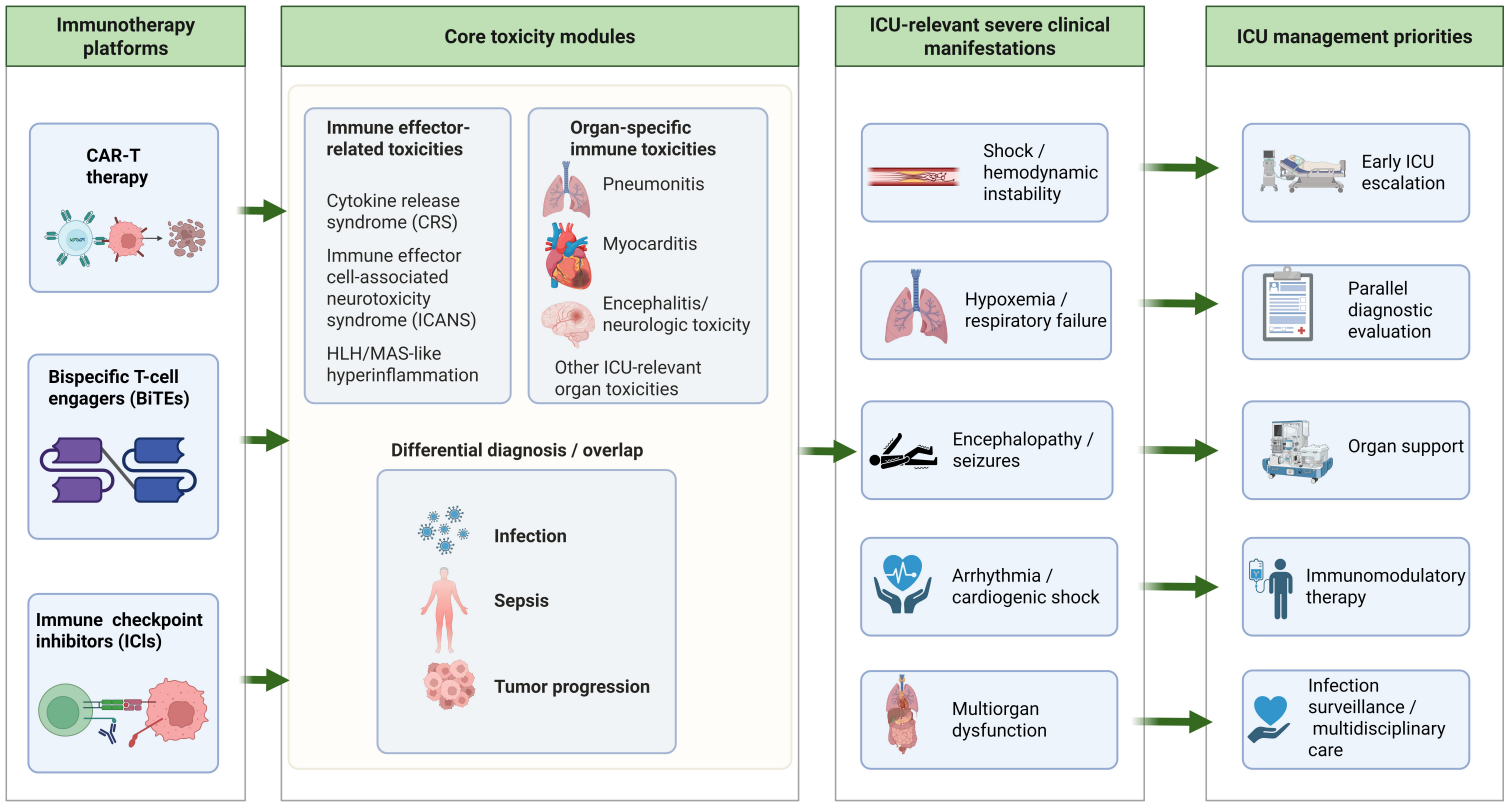
ICU-oriented diagnostic and management framework for immunotherapy-related critical illness. The algorithm contrasts the CAR-T/BiTE early inflammatory pathway with the ICI delayed organ-specific pathway and emphasizes parallel diagnostic evaluation, early organ support, empiric or targeted antimicrobial therapy when indicated, syndrome-directed immunomodulatory therapy, and dynamic multidisciplinary reassessment.

Taken together, these examples illustrate a broader principle: immunotherapy-related critical illness is best approached through parallel diagnostic reasoning rather than single-label classification. Initial working diagnoses should be treated as provisional and revised repeatedly as microbiology, imaging, laboratory trends, hemodynamic response, neurologic monitoring, and treatment effects become available (10, 14, 23, 31, 38, 55). The value of diagnosis in this field lies less in immediate precision than in supporting timely, safe, and adaptive management (25, 31, 57, 58). Table 2 outlines key bedside clues that favor immune-related toxicity, along with the most important diagnostic tests and immediate management priorities to guide ICU decision-making. Recognizing these overlaps and knowing when to escalate treatment is crucial in ensuring timely and effective care. Figure 2 provides an ICU-oriented diagnostic and management framework that contrasts the CAR-T/BiTE early inflammatory pathway with the ICI delayed organ-specific pathway and integrates parallel diagnostic evaluation, organ support, antimicrobial therapy, immunomodulatory treatment, and multidisciplinary reassessment.

**Table 2:** Major differential diagnostic conflicts in immunotherapy-related critical illness.

Clinical syndrome	Main competing diagnoses	Key bedside clues favoring immune-related toxicity	Priority investigations	Immediate management priorities	Common diagnostic pitfalls
CRS/hyperinflammatory shock	Sepsis; neutropenic infection; infusion reaction; adrenal crisis; tumor lysis syndrome	Recent CAR-T or BiTE exposure; typical early post-treatment timing; persistent fever with rapidly evolving hypotension and inflammatory deterioration; overlap with ICANS or known prior CRS trajectory	Blood cultures; chest imaging; urine culture; complete blood count; ferritin; CRP; procalcitonin; coagulation profile; liver and renal function tests; lactate	Hemodynamic support; early microbiologic sampling; empiric antimicrobials when infection cannot be excluded; tocilizumab and/or corticosteroids when clinically significant CRS is likely	Treating CRS and sepsis as mutually exclusive; delaying syndrome-directed therapy while awaiting culture results; overreliance on a single biomarker such as procalcitonin or ferritin
HLH/MAS-like deterioration	Severe sepsis; disseminated intravascular coagulation; progressive CRS; drug toxicity; acute liver failure	Rapidly rising ferritin; worsening cytopenias; coagulopathy; hypofibrinogenemia; progressive hepatic dysfunction; multiorgan failure despite initial CRS-directed management	Ferritin trend; fibrinogen; triglycerides; CBC; coagulation panel; liver function tests; microbiologic workup; bone marrow evaluation in selected cases	Escalated organ support; parallel infection coverage; early consideration of intensified immunomodulation when HLH/MAS is strongly suspected	Waiting for full formal diagnostic criteria before escalating treatment; mislabeling worsening immune dysregulation as persistent sepsis only; underestimating overlap with infection
ICANS/immune effector cell-associated neurotoxicity	Infectious encephalitis; sepsis-associated encephalopathy; metabolic encephalopathy; stroke; intracranial hemorrhage; medication-related delirium	Recent immune effector cell therapy; temporal association with CRS; early language disturbance, inattention, agraphia, or disorientation; fluctuating but progressive neurocognitive decline	Neurologic examination; EEG; brain CT/MRI; glucose and electrolyte testing; liver and renal function tests; lumbar puncture when safe and indicated	Airway protection when needed; seizure management; corticosteroids for clinically significant neurotoxicity; parallel exclusion of structural and infectious neurologic emergencies	Dismissing early language or attention changes as nonspecific delirium; delaying treatment while waiting for lumbar puncture or MRI; overlooking non-convulsive seizure activity
Immune-mediated encephalitis/related neurologic irAEs	Viral encephalitis; bacterial meningoencephalitis; paraneoplastic syndrome; brain metastasis progression; autoimmune CNS disease unrelated to therapy	Prior ICI exposure; subacute encephalopathy with inflammatory features; coexistence of other irAEs; absence of a more convincing infectious or structural explanation	Brain MRI; CSF analysis; EEG; infectious studies; autoimmune/paraneoplastic workup when appropriate	Early neurologic monitoring; empiric infectious coverage when indicated; corticosteroids if immune-mediated encephalitis is strongly suspected	Attributing all encephalopathy after immunotherapy to ICANS or to sepsis; failure to include ICI-related encephalitis in the differential because onset is delayed
Respiratory failure with suspected pneumonitis	Bacterial pneumonia; Pneumocystis jirovecii pneumonia; invasive fungal infection; viral pneumonitis; tumor progression; radiation pneumonitis; diffuse alveolar hemorrhage	Prior ICI exposure; compatible timing; bilateral inflammatory infiltrates without a dominant focal source; coexistence of other irAEs; worsening hypoxemia despite no clear infectious source	Chest CT; blood and respiratory cultures; fungal/viral testing; bronchoscopy/BAL in selected patients; CBC; inflammatory markers; oxygenation assessment	Escalating respiratory support; empiric antimicrobials when infection is plausible; corticosteroids when immune-mediated pneumonitis is strongly suspected; serial reassessment	Treating pneumonitis and infection as mutually exclusive; delaying corticosteroids until full microbiologic closure; overinterpreting CT pattern as diagnostic by itself
Respiratory failure after CAR-T/BiTE therapy	Infection; CRS-related lung injury; pulmonary edema; tumor progression; ARDS from nonimmune causes	Early post-treatment timing; concomitant CRS; diffuse inflammatory respiratory worsening in the context of systemic hyperinflammation	Chest imaging; blood cultures; respiratory sampling; hemodynamic and fluid assessment; ferritin and inflammatory markers	Oxygen or ventilatory support; hemodynamic optimization; empiric antimicrobials; CRS-directed therapy when clinically indicated	Attributing all respiratory deterioration to infection alone or to CRS alone; failing to recognize mixed pulmonary pathophysiology
Myocarditis/immune-mediated cardiac toxicity	Acute coronary syndrome; septic cardiomyopathy; stress cardiomyopathy; pulmonary embolism; drug-related arrhythmia	Recent ICI exposure; new conduction abnormality; unexplained troponin elevation; arrhythmia or syncope without a more convincing alternative explanation; overlap with myositis or neuromuscular symptoms	Serial ECGs; continuous telemetry; troponin and natriuretic peptides; echocardiography; cardiac MRI when feasible; coronary evaluation when clinically indicated	High-acuity cardiac monitoring; early corticosteroids when myocarditis is strongly suspected; arrhythmia management; pacing or circulatory support if needed	Waiting for severe ventricular dysfunction before considering myocarditis; assuming troponin elevation in the ICU is always ischemic or septic; delaying treatment until all cardiac investigations are complete
Shock in ICI-treated patients	Sepsis; cardiogenic shock from myocarditis; adrenal crisis; hypovolemia; pulmonary embolism	Prior ICI exposure; concurrent irAEs; unexplained hypotension not fully accounted for by infection or volume loss; endocrine or cardiac clues	Hemodynamic assessment; ECG; cardiac biomarkers; cortisol when relevant; echocardiography; infectious evaluation	Simultaneous hemodynamic stabilization, infectious workup, and evaluation for endocrine or cardiac immune toxicity	Framing shock as purely septic before considering myocarditis or adrenal insufficiency; delayed endocrine assessment in refractory hypotension
Acute liver dysfunction	Sepsis-associated cholestasis; ischemic hepatitis; drug-induced liver injury; tumor infiltration; biliary obstruction; HLH/MAS	Prior ICI exposure; other concurrent irAEs; inflammatory hepatic injury without a stronger obstructive or ischemic explanation	Liver panel; INR; bilirubin; abdominal imaging; viral hepatitis testing; medication review; ferritin and cytopenia assessment if HLH/MAS is suspected	Supportive care; evaluation for liver failure; corticosteroids when severe immune-mediated hepatitis is likely; treatment of competing causes in parallel	Labeling all liver test abnormalities as immune hepatitis; missing HLH/MAS, sepsis, or ischemic injury in the ICU
Acute kidney injury	Sepsis-associated AKI; hypoperfusion; tumor lysis syndrome; nephrotoxic drug injury; obstruction; immune-mediated nephritis	Prior ICI exposure for nephritis; CAR-T with CRS for multifactorial AKI; absence of a fully explanatory alternative cause; coexisting immune toxicity	Creatinine trend; urinalysis; electrolytes; renal ultrasound when indicated; medication review; hemodynamic assessment	Hemodynamic optimization; avoidance of nephrotoxins; renal replacement therapy when indicated; corticosteroids when immune nephritis is strongly suspected	Assuming all AKI in the ICU is hemodynamic; overcalling immune nephritis without considering shock, sepsis, TLS, or drugs

These diagnostic conflicts are often dynamic rather than binary. In critically ill patients, competing diagnoses may coexist, and initial management frequently needs to proceed in parallel before definitive diagnostic closure is achieved.

### Principles of ICU management

Management of immunotherapy-related critical illness differs from many traditional oncology ICU presentations not because basic critical care principles no longer apply, but because they must be integrated with time-sensitive syndrome recognition, immunomodulatory decision-making, and ongoing diagnostic uncertainty (2, 10, 23, 29, 51). High-quality care therefore depends on rapid stabilization, parallel etiologic evaluation, and early treatment of dominant threats before complete diagnostic closure is possible (10, 16, 23, 29, 51).

### Timing of ICU escalation

Early ICU escalation is one of the most important determinants of safe management in immunotherapy-related critical illness. These syndromes may worsen abruptly, and apparent physiologic stability can be misleading, particularly in patients with evolving encephalopathy, progressive inflammatory shock, worsening hypoxemia, or electrically unstable myocarditis (8, 9, 17, 30, 35). In practice, ICU transfer should be considered early when the patient demonstrates increasing oxygen requirement, persistent or escalating hypotension, rapidly changing mental status, seizure activity, new conduction abnormalities, dynamic troponin elevation, or evidence of worsening multiorgan dysfunction (9, 16, 17, 34, 66).

The purpose of ICU admission in this context is not merely to react to collapse after it occurs. It is to create the monitoring intensity, staffing, procedural capability, and organ support readiness needed to prevent potentially reversible syndromes from crossing into irreversible failure (8, 19, 30, 35, 36, 67).

### Organ support as a core therapeutic intervention

Organ support should be viewed as a central component of treatment rather than as a passive bridge to definitive diagnosis. In severe CRS, pneumonitis, ICANS, myocarditis, and other ICU-relevant toxicities, vasopressor support, invasive or noninvasive ventilation, renal replacement therapy, airway protection, seizure control, and temporary pacing are often the interventions that buy time for targeted anti-inflammatory therapy, antimicrobial treatment, and physiologic recovery to take effect (16, 17, 29, 30, 66).

This principle has practical implications. Respiratory failure should be managed with the same rigor used in other forms of critical lung injury (16, 23, 53), hemodynamic instability should be addressed promptly with vasoactive support and monitoring (8, 17, 30, 36), worsening encephalopathy should trigger airway assessment and neurocritical support (9, 33, 38, 59), and acute kidney injury should be interpreted as part of the broader physiologic trajectory rather than as a laboratory abnormality alone (2, 23, 47).

### Timing and escalation of immunomodulatory therapy

Although supportive care remains fundamental, syndrome-directed immunomodulatory therapy is frequently time-sensitive and may determine whether deterioration can be reversed. The need for early immunomodulation is most obvious in high-grade CRS, progressive ICANS, fulminant pneumonitis, myocarditis with electrical or hemodynamic instability, and hyperinflammatory syndromes evolving toward HLH/MAS-like physiology (8, 9, 16, 17, 32, 66). In such cases, waiting for complete diagnostic certainty may be more dangerous than treatment based on a well-supported working diagnosis (10, 16, 17, 23, 66).

The appropriate agent depends on the syndrome and treatment context. Tocilizumab and corticosteroids remain central in severe CRS (26, 29, 30, 36), whereas ICANS is more commonly managed with corticosteroid-based strategies and neurocritical support (9, 33, 38, 59). Severe checkpoint inhibitor pneumonitis and myocarditis generally favor early high-dose corticosteroids (16, 17, 23, 66, 68), while refractory hyperinflammatory states may require additional rescue therapies according to local practice and multidisciplinary input (10, 23, 29, 32).

### Infection surveillance and antimicrobial management

Infection surveillance should proceed in parallel with evaluation and treatment of immune toxicity, rather than after immune toxicity has been excluded (23, 29–31). Patients receiving CAR-T therapy, BiTEs, or ICIs may be vulnerable to infection because of malignancy-related immune dysfunction, prior chemotherapy, neutropenia, mucosal injury, central venous access, hypogammaglobulinemia, corticosteroid exposure, or additional immunosuppressive therapy (15, 23, 28, 31). Fever, shock, hypoxemia, or organ dysfunction may therefore reflect CRS, sepsis, immune-mediated organ injury, or a combination of these processes (8, 14, 29, 31, 38, 39).

Empiric broad-spectrum antimicrobial therapy should generally be started promptly after appropriate cultures are obtained in unstable patients, especially when fever is accompanied by hypotension, rising lactate, hypoxemia, altered mental status, profound neutropenia, focal infectious findings, central venous catheter concern, or ICU-level organ dysfunction (29–31, 39). In such patients, antimicrobial therapy and syndrome-directed immunomodulation should not be viewed as mutually exclusive (10, 23, 29, 31). For example, a CAR-T or BiTE recipient with suspected CRS and shock may require both antipseudomonal antibacterial coverage and CRS-directed therapy, while an ICI-treated patient with severe pneumonitis may require both infectious evaluation and early corticosteroids when immune-mediated lung injury is likely (29, 31, 38, 44).

Antimicrobial stewardship should rely on structured reassessment rather than withholding initial therapy in high-risk patients. Cultures, imaging, respiratory sampling, viral testing, fungal or Pneumocystis evaluation, and site-specific studies should be guided by the clinical syndrome and host risk (23, 29, 31, 39). If the patient stabilizes, cultures remain negative, no infectious source is identified, and the clinical trajectory supports immune toxicity, antimicrobial narrowing or discontinuation can be considered, often at 48–72 h, in collaboration with infectious diseases and oncology or hematology teams (23, 29, 31, 39). De-escalation should be more cautious in patients with persistent neutropenia, ongoing shock, progressive pulmonary infiltrates, prolonged corticosteroid exposure, second-line immunosuppression, or high risk for opportunistic infection (23, 28, 29, 31).

### Multidisciplinary coordination and structured pathways

Multidisciplinary coordination is essential because immunotherapy-related critical illness sits at the intersection of several forms of expertise. Intensivists must integrate physiologic stabilization and organ support; hematology or oncology teams provide treatment-specific context; infectious diseases specialists refine antimicrobial strategy; and organ-based consultants contribute syndrome-specific diagnostic and therapeutic guidance (3, 9, 23, 29). Institutions caring for high-risk immunotherapy populations therefore benefit from structured care pathways and early consultation triggers that reduce delay in recognition and treatment (29, 36, 41, 69).

### Goals of care and treatment boundaries

Decisions regarding treatment intensity should be individualized and should not rely on outdated assumptions about ICU futility in patients with cancer (19, 23, 41, 67). Severe immunotherapy-related toxicity may arise in patients whose oncologic trajectory remains potentially meaningful and whose acute syndrome is at least partly reversible (20, 34, 41, 67). Conversely, not every patient with treatment-related critical illness will benefit from maximal support (3, 19, 41, 67). ICU decision-making should therefore integrate the severity of current physiologic failure, reversibility of the suspected syndrome, underlying oncologic trajectory, pre-illness functional status, and patient-centered goals.

### A dynamic ICU framework

Taken together, ICU management of immunotherapy-related toxicity is best understood as a dynamic framework rather than a fixed protocol (23, 29, 41, 51). Initial treatment decisions must be made quickly and then revised continuously as microbiologic data, imaging, laboratory trends, organ support requirements, and response to therapy become clearer (16, 23, 29, 51). The core practical lesson is straightforward: act early, support aggressively, evaluate in parallel, and adapt continually. Table 3 summarizes the practical ICU management strategies for life-threatening immunotherapy-related complications, with a focus on timely escalation, organ support, immunomodulatory treatment, infection surveillance, and outcome-, follow-up-, and rechallenge-related considerations.

**Table 3:** Practical ICU management and follow-up considerations for life-threatening immunotherapy-related complications.

Syndrome/complication	Triggers for ICU escalation	Core organ support priorities	First-line immunomodulatory strategy	Escalation/rescue options	Key infection-related considerations	Major outcome/follow-up/rechallenge considerations
CRS	Persistent hypotension; escalating vasopressor need; increasing oxygen requirement; rising lactate; evolving multiorgan dysfunction	Hemodynamic support; oxygen therapy or mechanical ventilation; fluid strategy with close reassessment; renal replacement therapy when indicated	Tocilizumab for clinically significant CRS; corticosteroids in severe or progressive cases	Additional corticosteroid escalation; broader anti-inflammatory strategies in refractory inflammatory deterioration	Infection frequently coexists; cultures and empiric antimicrobials should often proceed in parallel	Outcomes are often favorable with early recognition and structured treatment. Persistent organ dysfunction should prompt reassessment for HLH/MAS or infection. For BiTEs, subsequent dosing may require delay, interruption, or modification according to toxicity grade and recovery.
HLH/MAS-like syndrome	Rapid ferritin rise; worsening cytopenias; coagulopathy; progressive liver dysfunction; refractory shock or multiorgan failure	Full ICU organ support; correction of coagulopathy; renal replacement therapy if needed; close reassessment of multiorgan dysfunction	Corticosteroids are central; management usually overlaps with severe CRS-directed therapy	Anakinra, etoposide, or other rescue immunosuppression depending on local practice and multidisciplinary input	Infection may mimic, trigger, or coexist with HLH/MAS; broad infectious evaluation and antimicrobial coverage are critical	Carries worse short-term prognosis than isolated CRS. Survivors require reassessment of organ recovery and future immunotherapy risk; re-exposure to the triggering platform should be highly individualized.
ICANS	Progressive encephalopathy; inability to protect airway; seizures; suspected cerebral edema; rapidly worsening ICE or equivalent neurologic assessment	Airway protection; seizure management; EEG monitoring; neurocritical care support; intracranial pressure-directed measures when needed	Corticosteroids are generally favored for clinically significant neurotoxicity	Escalated neurocritical support; intensified immunosuppression in refractory cases according to local expertise	Infectious encephalitis and sepsis-associated encephalopathy must remain in the differential; infection workup should proceed in parallel	Many patients recover, but severe or prolonged cases may leave neurocognitive sequelae. Future CAR-T or BiTE continuation/re-dosing should depend on severity, recovery, and product-specific guidance.
Immune-related pneumonitis/respiratory failure	Rapidly increasing oxygen requirement; severe hypoxemia; ARDS physiology; respiratory fatigue; need for high-flow oxygen, noninvasive ventilation, or invasive ventilation	Escalating respiratory support; lung-protective ventilation; prone positioning when appropriate; extracorporeal support in selected cases	Systemic corticosteroids for moderate to severe suspected immune-mediated pneumonitis	Second-line immunosuppression for steroid-refractory disease depending on local practice and severity	Empiric antimicrobial therapy is often appropriate initially; bronchoscopy/BAL may be useful when clinically feasible	Severe cases may have prolonged ventilatory needs and residual pulmonary dysfunction. Rechallenge after grade 3-4 or life-threatening pneumonitis is generally avoided; selected lower-risk patients may be considered only after clinical and radiographic recovery, steroid taper, and infection exclusion.
Immune-related pneumonitis/respiratory failure	Rapidly increasing oxygen requirement; severe hypoxemia; ARDS physiology; respiratory fatigue; need for high-flow oxygen, noninvasive ventilation, or invasive ventilation	Escalating respiratory support; lung-protective ventilation; prone positioning when appropriate; extracorporeal support in selected cases	Systemic corticosteroids for moderate to severe suspected immune-mediated pneumonitis	Second-line immunosuppression for steroid-refractory disease depending on local practice and severity	Empiric antimicrobial therapy is often appropriate initially; bronchoscopy/BAL may be useful when clinically feasible	Severe cases may have prolonged ventilatory needs and residual pulmonary dysfunction. Rechallenge after grade 3-4 or life-threatening pneumonitis is generally avoided; selected lower-risk patients may be considered only after clinical and radiographic recovery, steroid taper, and infection exclusion.
Respiratory failure after CAR-T/BiTE therapy	Worsening hypoxemia in the setting of CRS; diffuse lung injury; ARDS; hemodynamic instability with respiratory compromise	Oxygen therapy; invasive ventilation when needed; hemodynamic support; fluid balance optimization	CRS-directed therapy, including tocilizumab and/or corticosteroids depending on accompanying systemic toxicity	ARDS rescue strategies and selected extracorporeal support	Infection frequently overlaps with inflammatory lung injury; microbiologic evaluation and antimicrobials are often required in parallel	Outcome depends on CRS severity, infection burden, and timing of support escalation. For BiTEs, treatment delay or interruption may be considered after clinically significant respiratory toxicity.
Respiratory failure after CAR-T/BiTE therapy	Worsening hypoxemia in the setting of CRS; diffuse lung injury; ARDS; hemodynamic instability with respiratory compromise	Oxygen therapy; invasive ventilation when needed; hemodynamic support; fluid balance optimization	CRS-directed therapy, including tocilizumab and/or corticosteroids depending on accompanying systemic toxicity	ARDS rescue strategies and selected extracorporeal support	Infection frequently overlaps with inflammatory lung injury; microbiologic evaluation and antimicrobials are often required in parallel	Outcome depends on CRS severity, infection burden, and timing of support escalation. For BiTEs, treatment delay or interruption may be considered after clinically significant respiratory toxicity.
Myocarditis/cardiovascular toxicity	New conduction abnormality; malignant arrhythmia; syncope; rising troponin with instability; cardiogenic shock	Continuous telemetry; hemodynamic monitoring; pacing when needed; vasopressors/inotropes; mechanical circulatory support in selected cases	Early high-dose corticosteroids are generally favored in clinically high-risk presentations	Additional immunosuppression in refractory or fulminant cases; ECMO/MCS in selected patients	Infection and septic cardiomyopathy may coexist; parallel infectious and cardiac evaluation is essential	Short-term mortality remains high in fulminant cases; long-term cardio-oncology follow-up is required. Rechallenge with the same or another ICI is generally discouraged after severe myocarditis, especially with arrhythmia, conduction block, shock, or advanced cardiovascular support.
Immune-related encephalitis/severe neurologic irAEs	Worsening mental status; seizures; focal neurologic deficits; rapidly progressive CNS dysfunction	Neurocritical monitoring; airway protection when needed; seizure control; supportive care for raised intracranial pressure if present	Corticosteroids when immune-mediated CNS inflammation is strongly suspected	Additional syndrome-specific immunosuppression depending on local expertise	Infectious meningoencephalitis must be actively excluded; CSF and microbiologic testing remain important when safe	Recovery may be incomplete. Severe CNS toxicity generally makes future ICI rechallenge highly cautious and is often avoided unless oncologic benefit clearly outweighs neurologic risk.
Severe immune-related hepatitis	Encephalopathy; coagulopathy; rapidly rising bilirubin or INR; evolving acute liver failure; liver dysfunction as part of multisystem collapse	Supportive care for acute liver dysfunction; metabolic and coagulation management; hemodynamic optimization	Corticosteroids for severe suspected immune-mediated hepatitis	Additional immunosuppression for steroid-refractory disease depending on local practice	Must distinguish from sepsis-associated cholestasis, ischemic injury, viral hepatitis, drug toxicity, and HLH/MAS	Most mild cases are managed outside ICU. Severe or steroid-refractory hepatitis should make rechallenge cautious; any consideration requires biochemical recovery and exclusion of alternative liver injury.
Severe immune-related nephritis/AKI	Rapidly worsening kidney injury; refractory electrolyte abnormalities; severe acidosis; fluid overload; need for renal replacement therapy	Hemodynamic optimization; electrolyte and acid-base correction; renal replacement therapy when indicated	Corticosteroids if immune-mediated nephritis is strongly suspected	Additional immunosuppression in selected refractory cases	Sepsis, hypoperfusion, nephrotoxins, and tumor lysis must be considered in parallel	Renal recovery may be incomplete in severe cases. Rechallenge may be considered in selected patients only after renal recovery, nephrotoxin review, exclusion of alternative causes, and nephrology input.
Endocrine crisis, including adrenal crisis or diabetic ketoacidosis	Refractory hypotension; severe metabolic derangement; altered mental status; volume depletion	Hemodynamic stabilization; electrolyte correction; insulin and fluid management when appropriate; close metabolic monitoring	Syndrome-specific hormone replacement; corticosteroid replacement in adrenal crisis	Escalation of supportive care according to organ dysfunction	Infection may precipitate or coexist with endocrine crisis and should be assessed	Often reversible with prompt recognition. Once the crisis has resolved and stable hormone replacement is established, endocrine irAEs alone do not always preclude future immunotherapy.
Severe colitis/GI toxicity with ICU relevance	Shock; severe dehydration; perforation concern; major bleeding; sepsis	Volume resuscitation; correction of electrolyte derangements; surgical evaluation when indicated; ICU monitoring for sepsis and perforation	Corticosteroids for severe immune-mediated colitis when perforation and uncontrolled infection are not dominant	Additional immunosuppression for refractory disease according to local practice	Infectious colitis must be considered, especially in immunosuppressed patients	Severe, refractory, perforating, or ICU-level colitis may preclude rechallenge with the same regimen. Switching from CTLA-4-based therapy to PD-1/PD-L1 therapy may be considered only in selected recovered patients after gastroenterology and oncology input.

This table summarizes practical ICU management and follow-up considerations rather than disease-specific protocols. Rechallenge decisions should be individualized according to syndrome severity, organ recovery, steroid requirement, infection status, cancer benefit, available alternatives, patient preference, and multidisciplinary assessment. For ICI-related toxicity, decisions should distinguish rechallenge with the same agent from switching to a different checkpoint inhibitor class. CRS, cytokine release syndrome; HLH/MAS, hemophagocytic lymphohistiocytosis/macrophage activation syndrome; ICANS, immune effector cell-associated neurotoxicity syndrome; ICE, immune effector cell-associated encephalopathy; BiTE, bispecific T-cell engager; ICI, immune checkpoint inhibitor; irAE, immune-related adverse event; ARDS, acute respiratory distress syndrome; BAL, bronchoalveolar lavage; CNS, central nervous system; CSF, cerebrospinal fluid; ECMO, extracorporeal membrane oxygenation; MCS, mechanical circulatory support; AKI, acute kidney injury; GI, gastrointestinal.

### Heterogeneity across therapeutic platforms and patient populations

A central challenge in immunotherapy-related critical illness is that the same clinical syndrome does not carry the same meaning across all treatment contexts or patient populations (10, 19, 23, 70). Fever, shock, hypoxemia, encephalopathy, or myocardial injury may appear superficially similar at the bedside, yet the likely mechanism, pace of deterioration, reversibility, and management priorities may differ substantially depending on which immunotherapy platform was used, what underlying malignancy is present, and what physiologic reserve the patient brings to the ICU (17, 20, 23, 70).

### Therapeutic platform shapes the ICU phenotype

The immunotherapy platform is often the most informative early clue to the likely ICU phenotype. CAR-T therapy and BiTEs tend to generate acute syndromes dominated by systemic inflammation and neurotoxicity, especially CRS and ICANS (12, 22, 70, 71). ICIs, by contrast, more often produce delayed and organ-specific irAEs such as pneumonitis, myocarditis, encephalitis, colitis, or endocrine crisis (16, 17, 28, 34, 72). This difference changes ICU reasoning: in the immune effector cell setting, fever with shock early after treatment strongly raises concern for CRS, whereas after ICI exposure the dominant question is often which organ-specific irAE is being unmasked and how rapidly it is threatening reversibility.

### Oncologic context matters: hematologic malignancies vs. solid tumors

Underlying malignancy also changes the critical care meaning of severe toxicity (19, 20, 67, 70). Patients with hematologic malignancies, particularly those receiving CAR-T therapy or other intensive immune effector approaches, often enter the ICU with substantial infection risk, treatment-related cytopenias, prior transplantation exposure, and cumulative immunosuppression from multiple prior therapies (36, 47, 70, 73–75). Patients with solid tumors more often bring older age, baseline cardiopulmonary disease, prior radiation, major surgery, or chronic organ-specific impairment (34, 53, 72, 76). Accordingly, ICU severity reflects not only the toxicity itself, but also the narrow margin of compensation onto which that toxicity is imposed.

### Age and developmental context modify both recognition and recovery

Age further modifies both presentation and recovery. Pediatric patients receiving immune effector cell therapy may display trajectories and neurologic manifestations that differ from those of adults, while older adults may present with reduced reserve, more comorbidity, greater frailty, and slower functional recovery after critical illness (76–81). Age should not be used as a crude exclusion criterion for intensive care, but neither should it be treated as biologically neutral in discussions of benefit, risk, and recovery (76, 77, 81, 82).

### Host vulnerability may be more informative than broad category labels

Although treatment platform, cancer type, and age all matter, the most clinically useful predictor of ICU behavior is often not any broad category label but the specific pattern of host vulnerability present in the individual patient (73, 76, 77, 81–83). Baseline function, comorbidity burden, organ reserve, frailty, chronic immunosuppression, and cumulative damage from prior therapies often explain ICU trajectory better than taxonomy alone (71, 73, 74, 76, 77, 81–83). The most useful bedside question is therefore not simply what syndrome is present, but what that syndrome is likely to do in this patient over the next several hours.

### A heterogeneous field requires individualized critical care reasoning

The practical consequence of heterogeneity is that immunotherapy-related critical illness resists one-size-fits-all ICU reasoning (67, 70, 76, 81). Standardized frameworks remain valuable for communication and grading, but they must be interpreted through the lens of platform, malignancy, reserve, comorbidity, and evolving host vulnerability (70, 73, 81). Until better prognostic precision is available, heterogeneity should be treated as a central bedside fact rather than a footnote (70, 76, 81, 82).

### Outcomes, rechallenge, and unresolved questions

The growing ICU relevance of immunotherapy-related toxicity has made prognosis an increasingly important clinical question (20, 23, 67, 70). Yet outcome in this field cannot be reduced to a single metric (34, 67, 84, 85). Short-term survival, although crucial, captures only part of the consequence of severe toxicity (20, 34, 84, 85). Prognosis is better understood as a multidimensional process involving immediate survival, organ recovery, functional trajectory, and the implications of severe toxicity for future cancer treatment (84–86).

### Short-term outcomes: improving, but unevenly across syndromes

Short-term outcomes vary substantially across immunotherapy-related ICU syndromes and are shaped by both syndrome severity and timeliness of intervention (10, 19, 23, 70). In general, patients with isolated or promptly controlled CRS often have favorable short-term trajectories, whereas progression toward HLH/MAS-like physiology, refractory shock, severe multiorgan failure, fulminant myocarditis, or respiratory failure requiring prolonged mechanical ventilation is associated with substantially worse hospital outcomes (32, 66, 68, 70). Neurocritical complications occupy an intermediate position: many patients with ICANS improve with structured neurocritical care and corticosteroid-based treatment, yet severe cases may still be complicated by prolonged encephalopathy, status epilepticus, cerebral edema, secondary infection, and longer ICU stays (33, 38, 56, 59). From a critical care standpoint, the most useful prognostic question is often not which syndrome is present, but how much reversible physiology remains (23, 29, 67, 70).

### Survival is not the only outcome that matters

Hospital survival does not fully capture the burden of severe immunotherapy-related critical illness (34, 67, 84, 85). Survivors may remain vulnerable to persistent functional, cognitive, pulmonary, cardiac, endocrine, or treatment-related sequelae (85–87). Functional recovery, organ-specific sequelae, duration of immunosuppression, and return to cancer-directed therapy may be more clinically meaningful than survival alone, especially in older adults and in patients whose oncologic trajectory is otherwise favorable (81, 85–87). In this respect, the long-term consequences of severe pneumonitis, myocarditis, endocrine injury, multisystem irAEs, or prolonged immunosuppression may be highly relevant even when hospital discharge is achieved.

### Rechallenge after severe toxicity

Rechallenge after severe immunotherapy-related toxicity should be approached as a syndrome-specific and risk-adapted decision rather than as a uniform question of restarting treatment. In general, rechallenge may be considered only after substantial clinical recovery, resolution or stabilization of the relevant organ injury, tapering of corticosteroids to a low physiologic or near-physiologic dose when feasible, exclusion of uncontrolled infection, and multidisciplinary reassessment of cancer benefit vs. toxicity risk (63, 88–90). The decision should also distinguish between restarting the same agent and switching to a different checkpoint inhibitor class, because recurrence risk may differ according to the original toxicity, treatment mechanism, and available oncologic alternatives.

Certain toxicities should be viewed as high-risk or near-contraindications to rechallenge from an ICU perspective (66, 88–90). Severe ICI-associated myocarditis, especially when complicated by arrhythmia, conduction block, cardiogenic shock, mechanical circulatory support, or myocarditis–myositis–myasthenia overlap, generally argues against rechallenge (17, 23, 46, 66). Severe neurologic irAEs requiring ICU care should also prompt extreme caution. In contrast, selected patients with resolved pneumonitis, colitis, nephritis, hepatitis, or endocrine toxicity may be considered for rechallenge after individualized assessment, particularly when the initial event was not life-threatening, has fully improved, and no equivalent anticancer option exists (63, 88–90). For CAR-T therapy and BiTEs, the issue is often less “rechallenge” in the ICI sense and more whether subsequent dosing, re-dosing, or continuation after toxicity is safe; this should be guided by toxicity grade, response to CRS- or neurotoxicity-directed therapy, infection status, organ recovery, and product-specific guidance (26, 29–31).

### Why evidence remains limited

Despite rapid clinical adoption of immunotherapy, the evidence base guiding ICU prognosis and retreatment decisions remains limited. Much of the available literature comes from retrospective cohorts, single-center experiences, pharmacovigilance analyses, case reports, or expert consensus (68, 84, 89, 90). ICU-specific endpoints are often underreported, organ support intensity is inconsistently described, and distinctions between moderate toxicity and truly life-threatening critical illness are frequently blurred (34, 70, 84, 91). As a result, clinicians are often forced to extrapolate from oncology toxicity literature that does not fully capture the realities of intensive care (29, 84, 89, 91).

### Unresolved questions and future directions

Several unresolved questions define the next phase of this field. Better prognostic stratification is needed within ICU-level toxicity itself (23, 70, 84, 91); future work should integrate short-term ICU outcomes with longer-term recovery (67, 76, 84–87); and more precise data are needed to guide rechallenge and retreatment decisions (39, 68, 89, 90, 92). Above all, the field needs a stronger ICU-specific evidence base, including prospective registries, standardized reporting of organ support requirements and syndrome overlap, and closer integration between immuno-oncology and critical care research (70, 84, 91). Longitudinal datasets that link acute ICU physiology to durable organ sequelae, cancer treatment resumption, and long-term survival will likely be especially important (84–87).

### A more precise future challenge

The future of immunotherapy-related critical care will likely depend less on expanding syndrome lists and more on refining clinical precision: identifying which patients are likely to deteriorate abruptly, which remain most salvageable with aggressive support, which survivors are most likely to sustain durable sequelae, and which recovered patients can safely continue treatment (67, 70, 84, 85, 90). This future challenge is therefore not simply classificatory but prognostic and operational, requiring better tools for real-time risk stratification, better longitudinal outcome measurement, and more syndrome-specific evidence on retreatment safety (84–87, 89–94). In that sense, the next major advance in this field may come not from identifying new toxic syndromes, but from improving the precision with which ICU clinicians can predict reversibility, sequelae, and safe continuation of cancer therapy.

## Conclusion

Immunotherapy has transformed not only cancer treatment, but also the clinical meaning of critical illness in patients with cancer. In the ICU, this transformation is reflected in a new spectrum of syndromes that includes hyperinflammatory states, neurocritical complications, respiratory failure, cardiovascular emergencies, and other forms of immune-mediated organ dysfunction. These presentations differ from traditional oncology ICU patterns not simply because they are new, but because many of them are treatment-related, time-sensitive, and at least partially reversible when identified and managed early.

The central ICU challenge is therefore not merely recognizing the existence of these toxicities. It is determining, in real time, whether a patient's shock, hypoxemia, encephalopathy, myocardial injury, or multiorgan dysfunction is being driven primarily by immune-mediated toxicity, infection, tumor progression, conventional critical illness physiology, or a combination of these processes. Because these syndromes frequently overlap, diagnosis must be iterative and management must be parallel. High-quality care depends on timely ICU escalation, meticulous organ support, appropriately scaled immunomodulation, sustained infection surveillance, and structured multidisciplinary coordination.

At the same time, immunotherapy-related critical illness should not be approached with either reflexive pessimism or unqualified optimism. Many patients can survive these events and continue to derive oncologic benefit, which challenges older assumptions that ICU care in advanced cancer is inherently of limited value. However, outcomes vary substantially across syndromes and host contexts, and current evidence remains incomplete, particularly with respect to long-term recovery, rechallenge, and treatment optimization in the critically ill.

The future task is therefore one of refinement rather than justification. The field no longer needs only to establish that these patients may benefit from ICU care. It now needs better tools to identify high-risk trajectories earlier, distinguish overlapping syndromes more reliably, target treatment more precisely, and define recovery in ways that extend beyond short-term survival. As immunotherapy continues to evolve, so too must the ICU frameworks used to care for the patients it saves, complicates, and increasingly sends into critical care.
